# Building memory devices from biocomposite electronic materials

**DOI:** 10.1080/14686996.2020.1725395

**Published:** 2020-02-25

**Authors:** Xuechao Xing, Meng Chen, Yue Gong, Ziyu Lv, Su-Ting Han, Ye Zhou

**Affiliations:** a Institute of Microscale Optoelectronics, Shenzhen University, Shenzhen, P. R. China; b Institute for Advanced Study, Shenzhen University, Shenzhen, P. R. China

**Keywords:** Green electronics, biocomposite, data storage, resistive random-access memory, field effect transistors, 201 Electronics / Semiconductor / TCOs, 103 Composites

## Abstract

Natural biomaterials are potential candidates for the next generation of green electronics due to their biocompatibility and biodegradability. On the other hand, the application of biocomposite systems in information storage, photoelectrochemical sensing, and biomedicine has further promoted the progress of environmentally benign bioelectronics. Here, we mainly review recent progress in the development of biocomposites in data storage, focusing on the application of biocomposites in resistive random-access memory (RRAM) and field effect transistors (FET) with their device structure, working mechanism, flexibility, transient characteristics. Specifically, we discuss the application of biocomposite-based non-volatile memories for simulating biological synapse. Finally, the application prospect and development potential of biocomposites are presented.

## Graphical abstract

**Figure:**
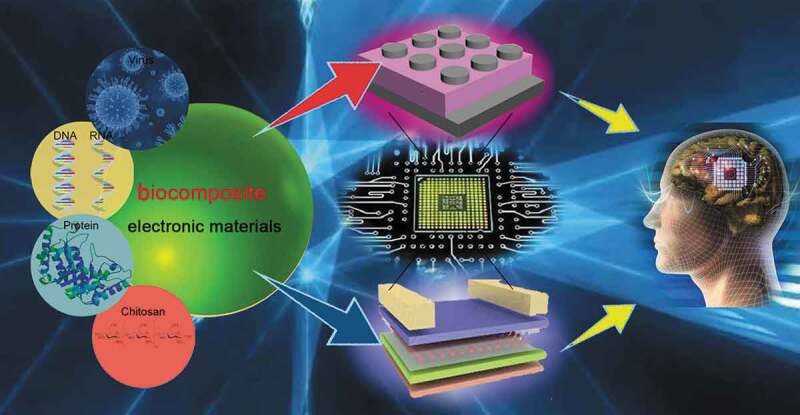

## 1. Introduction

With the wave of digitalization featuring big data, the internet of things (IOT), artificial intelligence (AI), and 5th generation mobile networks (5G) as core features, and the amount of data generated by humans is growing exponentially. Facing such a huge amount of data, in order to meet the needs of human data services, the production of electronic components is enormous, and the iterative update speed is fast [1–3]. What`s more, along with that, a large amount of electronic waste is generated. According to statistics, in 2016, 44.7 million tons of electronic wastes were generated worldwide, of which only 20 percent were collected and recycled properly, which not only causes a large amount of raw materials to be wasted, but also seriously pollutes the environment and even threatens our physical health. On the device level, the severity of this problem also requires us to rethink the way we design and manufacture devices [4–6]. In order to reduce e-waste, it is pretty significant for ‘green’ electronics to become mainstream technology; thus, environmentally friendly and biodegradable natural biological materials are the best choice to realize the sustainable development of the electronics industry. In addition, silicon-based traditional electronic storage devices need to face the von Neumann bottleneck problem, at the same time, trade-off in scalability, storage density, and cost efficiency is a challenge for traditional electronic devices [7].

Natural biomaterials not only can reduce the accumulation of hazardous waste, but also have the advantages of biocompatibility, flexibility, and cheapness, so that they have been widely used in bioelectronics, skin biosensors, medical biodiagnosis and so on [8–10]. However, biomaterials have a weak electron transfer function and are not compatible with traditional semiconductor device manufacturing processes; therefore, biomaterial-based degradable electronic products have many difficulties to overcome in order to achieve industrial feasibility [11–14]. Researchers have actively tried to improve the electrical function of composite materials through combining biological materials with various functional materials. In combination with other advantages, electronic devices based on biological composite materials are expected to compete with traditional silicon-based devices [15–17]. Recently, electronic devices based on biocomposite materials have been successively reported, which describe the development process of devices based on biomaterials, and fully reflecting the excellent characteristics including flexibility, transparency, stability and ecologically benign, among them mainly include two terminal-based resistive random-access memory (RRAM) and field effect transistors (FET) [18–21].

**Figure 1.**
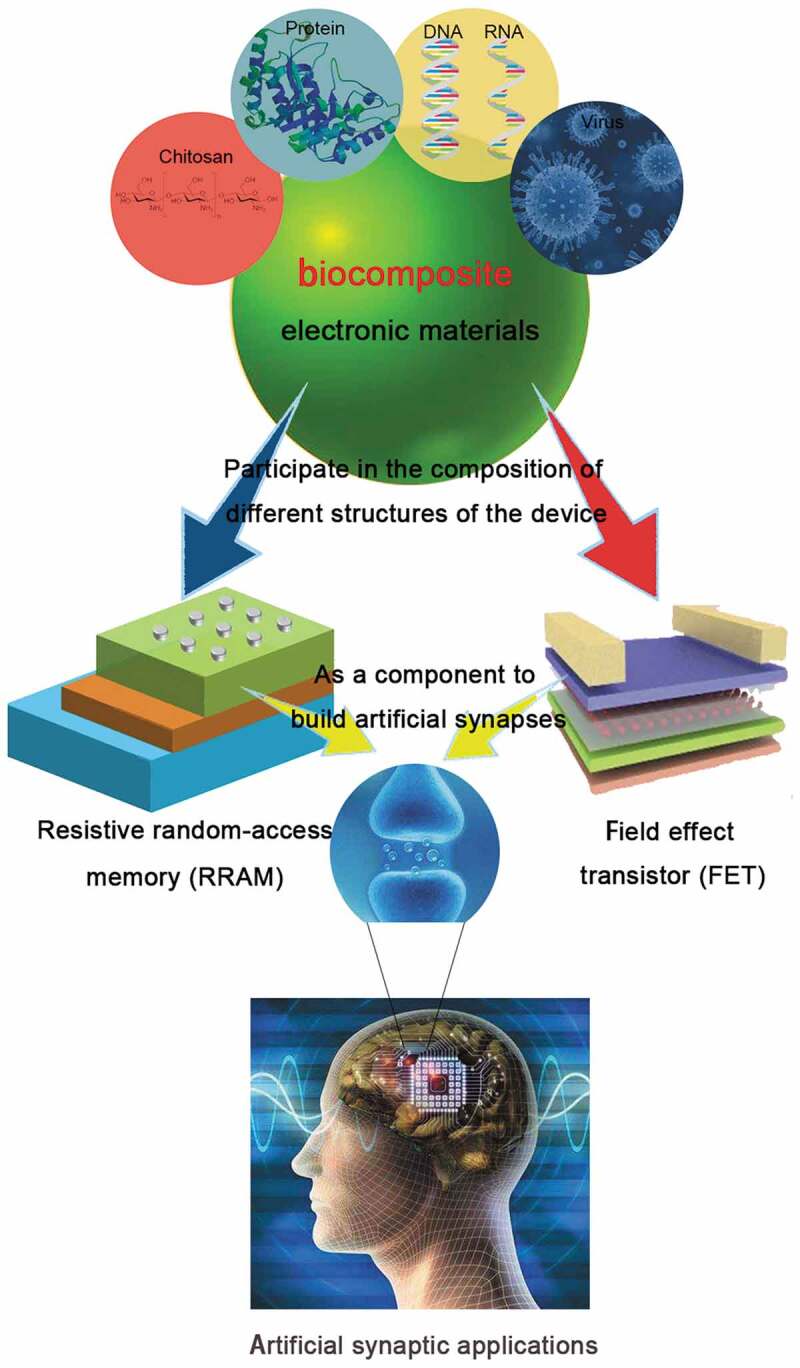
Outline of building memory devices from biocomposite electronic materials: different kinds of biocomposite materials will participate in the construction of memory devices with different structures and properties, and then the memory device will serve as a basic unit to simulate artificial synapses

**Figure 2.**
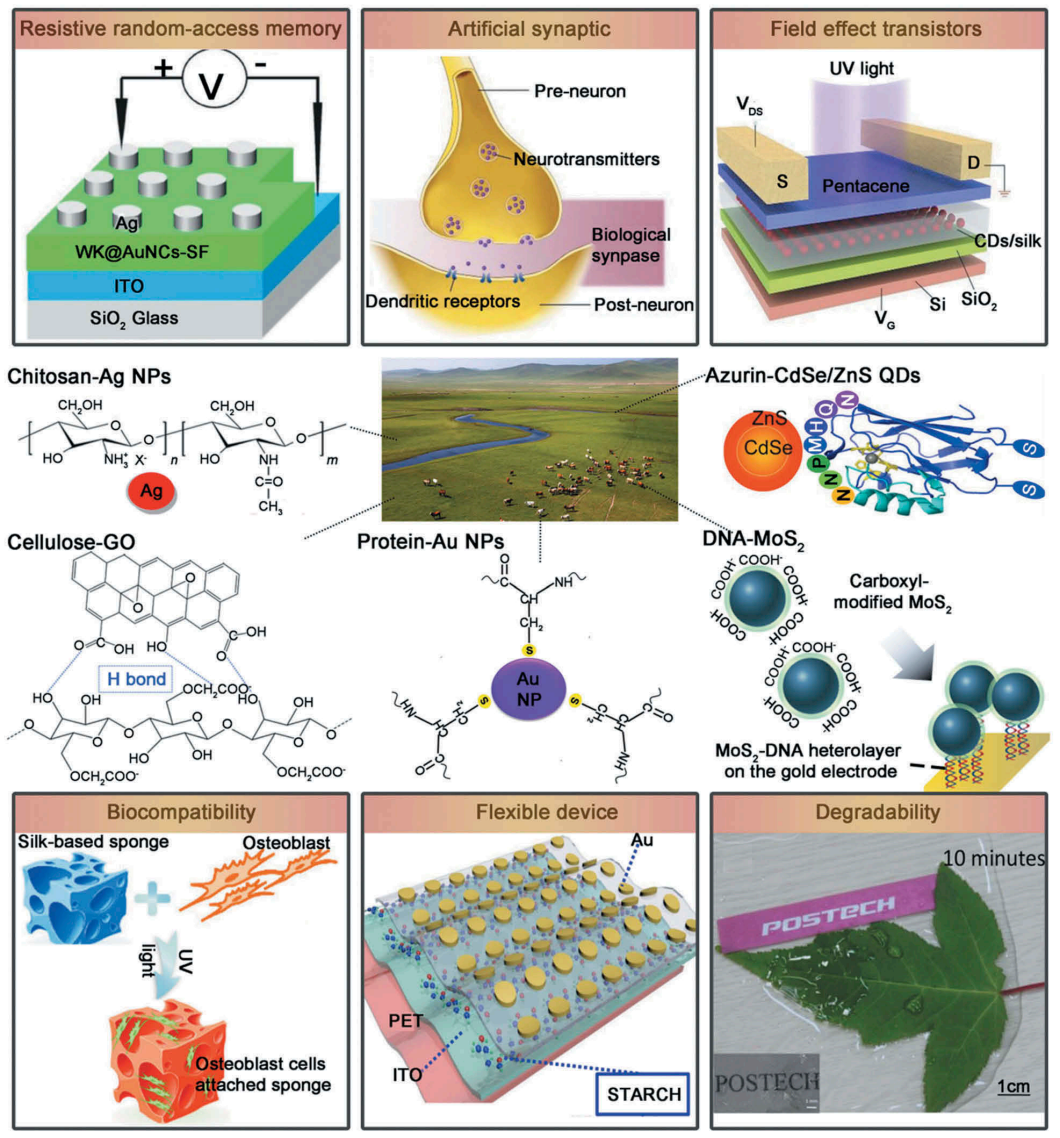
This review paper mainly introduces memory devices (RRAM and FET) composed of different biocomposites with biocompatibility, flexibility and biodegradability. On this basis, the progress of artificial synapses to mimic biological synapses is discussed and the future development of green electronic devices is presented. Reprinted with permission from [28]. Copyright 2017 WILEY‐VCH; [44]. Copyright 2018 WILEY‐VCH; [48]. Copyright 2015 WILEY‐VCH; [53]. Copyright 2016 Elsevier B.V.; [57]. Copyright 2019 Elsevier B.V.; [58]. Copyright 2019 Elsevier Ltd.; [64]. Copyright 2016 American Chemical Society; [83]. Copyright 2019 WILEY‐VCH]

In this review, the introduction can be roughly summarized as follows (Figure 1): Firstly, we will mainly summarize and discuss the application of biological composite material in the electronic device such as RRAM and FET. After introducing the fundamentals in non-volatile memory, we will specifically focus on a variety of biological composite material in RRAM. Thereafter, we will discuss the biological composite material of FET with novel properties, and then introduce the artificial synapses based on biological composite material, which shows the bionic memory device in terms of innovation and progress. Finally, we will summarize and forecast the development trend of biocomposite devices, and expect that biocomposite electronic devices can accelerate the development towards ‘green’ electronics. In addition, Figure 2 shows various types of biocomposites in recent years. Based on these biocomposites, memory devices with special properties were fabricated, and artificial synapses were simulated using these memory devices.

## 2. Biocomposite for memory based on resistive structure

### 2.1. RRAM

Since the birth of the first computer, the structure of the modern computer system is still based on the von Neumann principle, which is mainly composed of five parts: memory, arithmetic, controller, input equipment and output equipment. Among them, memory is used to store all kinds of data, which is an indispensable part of the computer. In addition, memory can be divided into volatile memory and non-volatile memory according to the data storing time in the memory. Volatile memory, where information is lost after a power outage, is mainly used to store programs that are used for a short period of time, such as dynamic random access memory (DRAM) [22–24]. The non-volatile memory can retain stored information after switching off the power, such as flash memory. With the popularization of mobile phones, digital cameras and other portable electronic devices, non-volatile memory is playing an increasingly important role in modern human’s daily life. With progresses of science and technology, the application of new non-volatile memory will bring a qualitative change to the performance of computers and people’s operating habits. The International Technology Roadmap for Semiconductor (ITRS), released in 2013, recommends RRAM after classifying and evaluating new types of memory currently being studied, and recommends acceleration of the commercialization.

Generally speaking, RRAM is a metal/insulating layer/metal (MIM) unit structure device, and due to its simple structure, it can be expanded into a three-terminal or four-terminal device, facilitating large-scale integration and high-density storage through the cross-array structure. For its device structure, the metal (M) can be any good electronic conductor, and the insulating (I) layer is a functional material with memristive properties, such as oxide, organic compound, and so on. The resistance transition effect, as its name implies, refers to the phenomenon that the resistance of a material change under the action of an electric field [25]. Materials with resistance transition characteristics exhibit a hysteresis I-V curve under the action of DC voltage, and this change in resistance usually does not deteriorate with time. However, the resistance transition characteristics observed in different material systems are different. Based on their typical I-V curves, the resistance transition behavior can be divided into two categories: unipolar resistance transition and bipolar resistance transition. In the unipolar transition, the resistance transition depends on the amplitude of the applied voltage rather than the polarity of the voltage, and the transition between high and low resistance states can be achieved under both positive and negative voltages [26]. When the original state device is in the high-resistance state (HRS), it enters low-resistance state (LRS) after applying a high voltage, and then a lower voltage value is applied after the transition to LRS, resulting in the transitions from LRS to HRS. Generally, the process of changing the device from HRS to LRS is known as the SET process, and the process of transferring the device from LRS to HRS is called the RESET process. Furthermore, the bipolar resistance transition phenomenon is related to the polarity of the voltage, the SET process can occur only when the device is excited by a voltage of a specific polarity, and the RESET process can occur when a voltage in the opposite direction is applied [27 28,].

At present, due to the wide range of resistance switching material systems, there is no unified theory that can explain all the resistance switching (RS) behaviors. In general, the RS mechanism can be divided into: electrochemical metallization mechanism (ECM), valence change mechanism (VCM), thermochemical mechanism (TCM) [29]. ECM usually appears in devices with active metals as electrodes, typically the device is initially in HRS, when the metal ions generated by the electrode under the action of voltage are moved to the inert electrode in the functional layer material and reduced to conductive filament (CF) in the film, the device turned to LRS. Then, under the reverse operating voltage, CF dissolves and the device returns to HRS [30 31,]. In VCM, most of the CF is formed by the migration of oxygen ions, but the oxygen ions will accumulate at the interface between the electrode and the functional layer, leading to change in the barrier height and thus affect the switching process [32 33,]. Therefore, the RS mechanism of some devices is often determined by VCM and interface barriers, and TCM usually occurs in unipolar RRAM, the formation and diffusion of CF are dominated by thermal effects [34]. In recent years, RRAM has been considered as an ideal device for simulating synapses due to its non-volatile nonlinear conduction characteristics, and it is possible to break the von Neumann bottleneck. Voltage-gated ion channels have similar properties to tunable conductance and can be able to play a key role in the construction of future neural networks [25 35,].

### 2.2. RRAM based on metal nanoparticle-doped biomaterials

RRAM based on organic materials is a burgeoning non-volatile memory with advantages of low power consumption, compatibility, reliability, high switching ratio, high storage density and fast response time. Recently, a lot of polymers are used for RRAM with non-volatile properties [36]. Notably, biomaterials are considered as the most suitable material to realize the emerging generation of ‘green’ electronic products on account of their unique biological structure, perfect functions, biocompatibility, excellent flexibility and biodegradability. Liu et al. reported that flexible RRAM based on egg albumen as switching layer with long retention time and fast switching speed. Its switching mechanism can be attributed to the formation and dissolution of dispersed Ag nanoclusters in the albumen membrane through the redox reaction of the Ag electrode [37]. However, the electron transfer function of numerous natural biomaterials is not particularly outstanding, and its actual performance in non-volatile memory devices needs to be further improved. Based on this, numerous researchers have proposed hybrid memory technology based on biomaterials based composites for non-volatile resistive memory elements [38–41]. So far, biomaterials have been hybridized with various materials and obtained excellent performance; we will describe the representative and progressive research and the performance of biomaterial doped materials-based will also be counted (Table 1).

**Table 1.** A performance comparison of biocomposite-based RRAM

Biocomposite unit	Device structure	Device type	Set/Reset voltage	ON/OFF ratio	Cycle number	Retention time	Ref
Pt NPs-tobacco mosaic virus (TMV)	Al/TMV–Pt NPs/Al	RRAM	3/–2.4 V	10^3^	400	–	[16]
Au NPs-silk	Al/silk-Au NPs/PET	Flexible RRAM	2/–2 V	10^4~^10^6^	10	10^3^ s	[42]
Au NPs-sulfhydryl groups	Al/Albumen layer/Pt	RRAM	2/–1.5 V	30	100	10^4^ s	[44]
Au NPs-alkali lignin	Al/Au NPs-alkali lignin/Al	FlexibleWORM	4.7 V	>10^4^	–	>10^3^ s	[45]
Ag-cellulose nanofiber paper (CNP)	Ag/Ag-CNP/Pt Ag/Ag-CNP/Al foil	Flexible RRAM	0.28/–0.22 V	10^6^	100	10^5^ s	[46]
Ag-chitosan	Pt/Ag-chitosan/Ag	RRAM	0.4 V/–0.48 V	10^5^	100	10^4^ s	[47]
Ag-chitosan	Mg/Ag-chitosan/ITO-coated PET	Flexible RRAM	1.63/–0.82 V	>10^2^	60	10^4^ s	[48]
Azurin-CdSe/ZnS QDs	Azurin-CdSe/ZnS/Au	RRAM	2/0.3 V	10^3^	50	–	[53]
Silk fibroin-CdSe QDs	Al/CdSe-CF/ITO	Multilevel RRAM	0.17/0.03 V	10^3^	50	10^4^ s	[54]
Carbon dots (CDs)-silk protein	Ag/CDs-silk/ITO	RRAM	0.5/–0.9 V	>10^6^	100	10^6^ s	[56]
MoS_2_ -DNA	MoS2 -DNA/Au	RRAM	2.4/0.01 V	–	–	10 days	[57]
Carboxymethyl cellulose-graphene oxide	Al/CMC-GO/Al	Flexible RRAM (WORM)	2.22 V	10^5^	–	>10^4^ s	[58]
DNA- cetyltrimethylam- monium (CTMA)	Ag/DNA-CTMA/ITO	RRAM (WORM) (WREM)	1 ~ 4/–2~-9 V	–	200	>10^4^ s	[62]
Starch−chitosan	Au/starch−chitosan/ITO-PET	Flexible RRAM	0.9/–1.6 V	>10^2^	200	>10^4^ s	[64]
Wool keratin (WK)@AuNCs- Silk Fibroin	Ag/WK@AuNCs-SF/ITO	RRAM	0.3/–0.3 V	10^2^	–	>10^4^ s	[28]

#### 2.2.1. Hybridization of Au/Pt NPs and biomaterials

**Figure 3.**
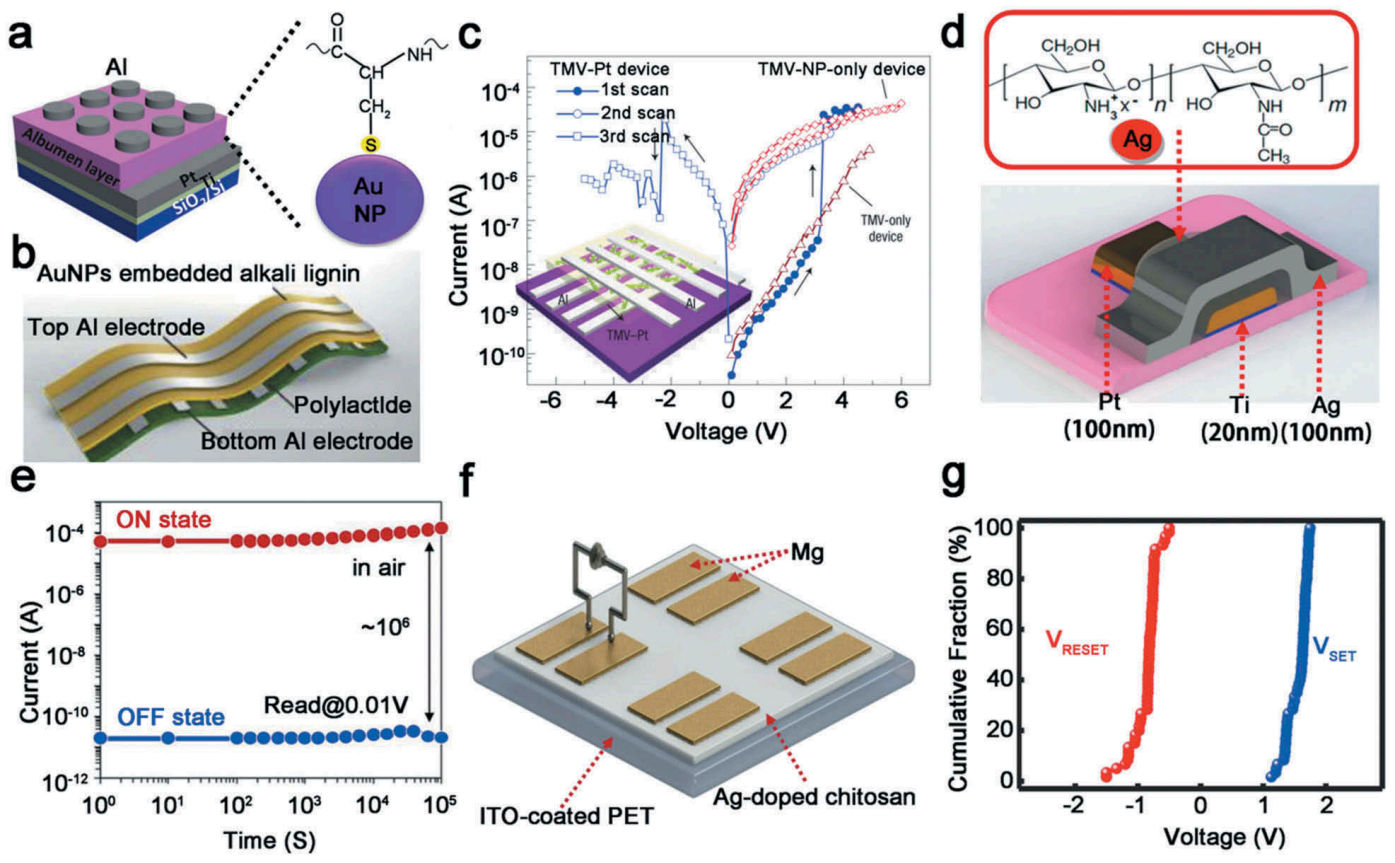
Device structure and electrical properties of RRAM based on metal nanoparticle-doped biomaterials. (a) Schematic illustration of Al/Albumen-Au NPs/Pt device and Au NPs and bonding of Au NPs to sulfhydryl groups of albumen. Reprinted with permission from [44]. Copyright 2018 WILEY‐VCH. (b) Schematic flow chart of the fabricated alkali lignin-based flexible memory device. Reprinted with permission from [45]. Copyright 2018 Elsevier B.V. (c) Typical I-V curve of the TMV-Pt device﻿; the inset shows schematic of TMV-Pt device structure. Reprinted with permission from [16]. Copyright 2006 Springer Nature. (d) Device configuration of RRAM based on Ag-doped chitosan and structure of chitosan chemical. Reprinted with permission from [47]. Copyright 2015 American Chemical Society. (e) Retention time of the Ag-decorated CNP device. Reprinted with permission from [17]. Copyright 2014 Springer Nature. (f) Schematic of the device with Mg/Ag-doped chitosan/Mg structure. (g) Set/reset voltage statistical distribution of Mg/Ag-doped chitosan/Mg devices. Reprinted with permission from [48]. Copyright 2015 WILEY‐VCH

Owing to its unique selectivity, nanomaterials have become an ideal biological detection system, and the synthesis of biological nanostructures using nanocrystals has made important progress. However, the use of soft materials as templates to organize inorganic nanoparticles is critical for building new functional electronic devices, few of which are made directly from biological materials due to lack of charge transfer. Hybridization with metal nanoparticles can improve the conductivity of biomaterials, reduce the randomness of conductive path growth effectively, and improve the robustness, uniformity and storage performance of device. In 2006, Yang et al. reported a memory based on the hybridization of tobacco mosaic virus (TMV) and platinum nanoparticles (Pt NPs), which had the characteristics of non-volatile memory and high current switching ratio (10^3^) (Figure 3(c)). The author hybridized TMV and Pt NPs in a solution of platinum ions, and platinum ions were evenly distributed on the surface of the virus by binding to the functional groups of proteins. In this bistable device, the TMV not only controls the arrangement and size of Pt NPs, but also provides charges for the charge transfer between nanoparticles, sequentially achieving the data storage function. This suggests that the electric field can stimulate the charge transfer from the virus to the Pt NPs, thereby enhancing the electron transfer after hybridization in the biomaterial [16].

Silk protein has become one of the most widely used biocompatible materials in the application of tissue engineering, regenerative medicine and other biotechnologies because of its mechanical robustness, flexibility in the form of thin films, optical transparency and compatibility with water treatment. In 2013, Gogurla et al. demonstrated a flexible resistive memory comparable to traditional metal oxide memories, in which Au NPs/silk protein fibers composite acted as an intermediate layer. During the setting process, a large number of negatively charged Au NPs would gather near the electrode where a positive voltage was applied, and form a conductive path with the oxidized silk protein between the two electrodes. In contrast, during the reset process, the electrodes repelled Au NPs, leading to the destruction of the conductive pathways and the reduction of silk proteins. Since the lower bias voltage will generate a larger electric field at the tip of Au NPs, the set/reset voltage (2 V) of Au NPs hybrid devices was much lower than that of monofilament devices, besides the switching ratio has reached 10^6^. What is more, the HRS and LRS were evenly distributed, which showed that the incorporation of Au NPs into silk fibroin not only improved device performance, but also helped achieve multi-level storage and low power consumption of protein-based RRAM [42].

However, the disadvantage is that in the process of protein fusion with Au NPs, the excessive aggregation of Au NPs may lead to uneven size, which affects the success rate and affect the stability of the device. Therefore, it is critical to form a uniform conductive path in the device by ensuring the size uniformity of the Au NPs. Uraoka’s team studied how to control the uniformity of doping metal nanoparticles in the RRAM insulating layer. Interestingly, although the RRAM insulation they reported was not a biomaterial, they controlled the size of the metal nanoparticles through a cage-shaped ferritin cavity with an outer diameter and an inner diameter of 7 nm and 12 nm, respectively. Due to the formation of controlled conductive paths, the Pt NPs-doped devices showed stable unipolar resistive switching behavior and uniformly distributed high-impedance state, but the switching rate was only 10^2^. From this point of view, how to further improve the uniform distribution of dopants in biomaterials is a target [43]. In 2018, Lee et al. demonstrated that poly(vinylpyrrolidone) (PVP) could encapsulate Au NPs to prevent large-scale agglomeration of Au NPs in protein solutions, and the feasibility of this process was confirmed using UV-visible spectroscopy. The authors mixed PVP-coated Au NPs and Au NPs with protein solution, respectively. At the beginning, the material showed the same absorption peak (530 nm), but when the time continued for 12 h, the protein solution doped with Au NPs became purple; meanwhile, the absorption peaks shifted, indicating that Au NPs have condensed on a large scale. The authors clarified that the aggregation of Au NPs is due to the spontaneous formation of Au-S bonds between Au and sulfhydryl groups in the protein solution (Figure 3(a)). This reaction will lead to the loss of negative charge of Au NPs, further reducing the electrostatic repulsion of Au NPs and causing easier aggregation, which will lead to an irreversible conversion of the device and exhibit write-once read-many memory (WORM) characteristics. However, the absorption peak of the protein solution of Au NPs covered by PVP remained unchanged, that indicating the cap layer of PVP prevented the aggregation of Au NPs and the device shows the feature of normal RRAM. Finally, the author concluded that the migration of electrons along the trap positions formed by Au NPs is the main reason for the device to exhibit RS behavior [44]. In the same year, Roy et al. proposed a similar situation. They reported that the memory based on Au NPs doped into alkali lignin also exhibits typical WROM behavior, which is flexible and degradable (Figure 3(b)). Moreover, the on/off current ratio of the device is as high as 10^4^, and it shows good reliability and robustness [45].

#### 2.2.2. Hybridization of Ag NPs and biomaterials

In addition to incorporate inert Au, Pt nanoparticles into biological materials, Ag is also quite popular as a dopant, especially in flexible devices, and the formation and fracture of Ag CF in the resistive switching layer has been researched a lot. In recent years, flexible memories based on Ag and biomaterials have shown superior device performance in terms of switching ratio and set/reset voltage distribution. In 2018, Chang et al. used a gelatin film embedded with Ag as an insulating layer to fabricate an RRAM device with highly uniform resistance switching characteristics [46]. The dissolved AgNO_3_ in the gelatin solution ensured the uniform distribution of Ag in the gelatin and promoted the local orientation of the growth of the CF, which is the reason for the good distribution of the switching voltage. It is worth mentioning that the set/reset voltage distribution of this device is relatively concentrated, and the on/off current ratio exceeds 10^5^. Cellulose is the oldest and most abundant natural biopolymer on the earth, and is an inexhaustible and inexhaustible renewable resource, which has increasingly aroused research interest on biomaterial-based RRAM devices. In 2014, Nagashima and others used Ag-modified cellulose nanofiber paper (CNP) to demonstrate a memory with ultra-flexible non-volatile performance. The on/off current ratio changed by 10^6^ in Ag-modified CNP RRAM (Figure 3(e)), which showed good robustness and data storage performance although with a bending radius of 350 mm. Therefore, these results highlighted the potential usage of Ag as a dopant to modify flexible biomaterials in electronic data storage device [17].

In general, the amino group in the molecular structure of chitosan has strong reactivity, which endows polysaccharide with versatile biological functions and can be chemically modified. Based on this, Lee’s group in 2015 reported a Pt/Ag-doped chitosan/Ag flexible RRAM that demonstrated good repeatability and reliable bipolar resistance switching characteristics (Figure 3(d)). In addition, the device not only had excellent non-volatile data storage performance, but also showed a high on-off current ratio (up to 10^5^) and long retention time (10^4^ s). By measuring the conductive properties, the author believed that the formation and fracture of the Ag CF was the reason why the device exhibited repeatable resistance switching behavior. Furthermore, this phenomenon has been widely used to explain the mechanism of bipolar RRAM and provides a new development direction to Ag-doped biomaterials in biocompatible and flexible memory devices [47]. At the same year, in order to further improve previous research results, Lee’s group used the metal Mg with high conductivity and biodegradability as the electrode to manufacture a fully biodegradable non-volatile memory device with Mg/Ag-doped chitosan/Mg structure on a transparent flexible substrate (Figure 3(f)). While maintaining a long retention time of 10^4^ s and a stable set/reset voltage (Figure 3(g)), the device also showed outstanding flexibility. The experimental results also depicted that the chitosan-based memory has advantages of good degradability, low cost and low power consumption. Moreover, the vacuum-free solution method described in this paper is also used many times in subsequent material doping. Overall, for the development of flexible memory with biomaterials and biodegradable devices, Lee’s work is an important step forward [48]. Afterwards, in 2018, Strehle et al. reported the resistance switching behavior of Ag-doped chitosan in different proportions. For a 0.5 wt% chitosan-doped film, the LRS can be maintained for a long time (8 days) with a small voltage range (2V) [49].

In this section, resistance switching memories of biomaterials with doped metal nanoparticle have been summarized. Doping of metal nanoparticles can compensate the conductive properties of biomaterials and make full use of their flexibility, biodegradability and biocompatibility. In addition, biomaterials based on Fe ions [39], Cu ions [50] and Ni ions [51] can also be used as insulation layers to develop flexible degradable resistance switch memories.

### 2.3. RRAM based on semiconductor material doped-biomaterial

The above research also has certain limitations, such as narrow bistable voltage range and poor stability. In order to overcome these problems, excellent semiconductor materials and stable insulating materials are strongly recommended. To date, various insulating or semiconductor materials, such as quantum dots (QDs), two-dimensional transition metal sulfide (TMDs), graphene and its derivatives, and binary transition metal oxides, have demonstrated excellent resistance switching properties. Among these nanomaterials, QDs are often used as dispersion in organic matrices to increase electrical conductivity, resulting in bistable memory. Specifically, core-shell QDs range in diameter from 1 nanometer to tens of nanometers, and their outer layers are lidded ligands that are chemically modified to carry large numbers of functional groups attached to biomolecules. The binding of biomolecules and QDs is achieved by forming covalent bonds with different linkers, molecules and QDs self-assemble under electrostatic interaction. In recent years, two-dimensional (2D) materials have attracted the interest of the scientific community because of their excellent performance and potential device applications. For 2D materials, carriers can only move along the direction of thickness, and they have a large plane area and a high sensitivity to the outside environment, which is an important characteristic suitable for a variety of applications [52]. Therefore, hybrid structures containing nanomaterials are becoming potential applications for the next generation of non-volatile data storage devices.

#### 2.3.1. RRAM based on QDs-doped biomaterials

Semi-conductor nanoparticles, especially quantum dots (QDs), are often used in the fields of biomarkers, medicine, sensing, information transmission and data storage due to their excellent performance, photoelectric effect, size adjustability and doping uniformity. In addition, QDs-doped biomaterial RRAM points the way to a new paradigm for electronic skin with flexible degradability. Here, we will discuss the performance of different QDs-doped biomaterials based RRAM.

**Figure 4.**
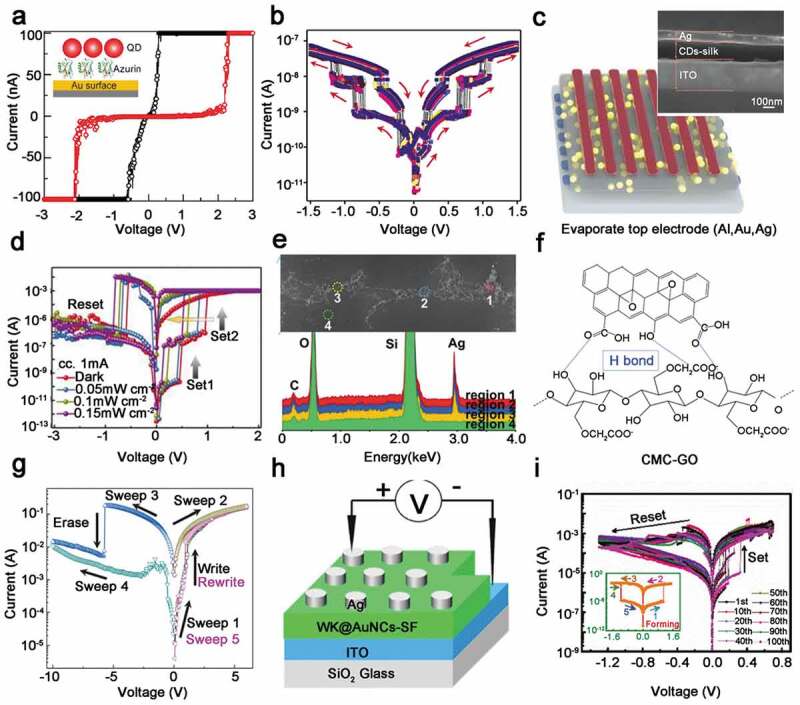
Device structure and test performance of RRAM based on semiconductor material doped-biomaterial. Reprinted with permission from [53]. Copyright 2016 Elsevier B.V. (a) Typical I-V curves of the QDs-Azurin/Au device; the inset diagram is device structure. (b) Schematic diagram of multi-level storage characteristics of a device with an Al/CdSe QDs: SF/ITO device under 50 cycles. Reprinted with permission from [54]. Copyright 2017 Elsevier B.V. (c) Schematic of the device with mental/CDs-silk/ITO structure; inset is cross-section SEM imaging of the device. (d) I-V characteristics of Ag/CDs-silk/ITO devices under different UV light intensities. (e) SEM imaging of Ag CF and energy-dispersive spectroscopy analysis of different parts. Reprinted with permission from [56]. (f) The bonding diagram of CMC-GO composite. Reprinted with permission from [58]. Copyright 2019 Elsevier Ltd. (g) DNA biopolymer-based device achieves WREM characteristic curve under light regulation. Reprinted with permission from [61]. Copyright 2015 AIP Publishing. (h) Schematic of the RRAM device with WK@AuNCs-SF as resistive layer. (i) I-V characteristics of Ag/neat-SF/ITO device (illustration is I-V curve of Ag/WK@AuNCs-SF/ITO device). Reprinted with permission from [28]. Copyright 2017 WILEY‐VCH

QDs are semiconductor nanostructures that bind excitons in three spatial directions, with size-controlled emission spectra and electronic properties. In 2017, Choi et al. reported an RRAM device based on recombinant azurin-CdSe/ZnS hybrid structure [53]. As mentioned earlier, metal nanoparticles of biomaterials interact via strong chemical bonds or electrostatic forces, and hence the nanoparticles do aggregate. The author overlaid ZnS on CdSe QDs to form core-shell QDs. ZnS inorganic shell acts as a barrier for charge transfer between adjacent QDs, so that CdSe-ZnS QDs have stronger robustness and better distribution uniformity during hybridization. What is more, the experimental results showed that in the repeated 50-times I-V tests, the switching current ratio remained at 3 orders of magnitude and the operating voltage was stable at about 2V (Figure 4(a)), manifesting the good bipolarity. Subsequently, in another report, the author conducted a detailed study on RS mechanism. There was no obvious bipolar resistance switching behavior in CdSe-ZnS/Au device and azurin/Au device, so the author believed that the bipolar behavior was caused by charge transfer between protein materials and core-shell QDs. Among resistive switching memories, hybrid materials are often used as a single insulating layer to study the performance of memories. In 2017, Murgunde and Rabinal reported that the silk fibroin (SF) integrated with CdSe QDs was spin-coated separately to make Al/CdSe QDs: SF/ITO devices, where ITO stands for indium tin oxide, in which SF and CdSe QDs formed a double-layer insulation floor [54]. Through positive and negative scanning, the devices showed superior multi-level data storage capabilities, and each level of memory window can reach an order of magnitude with low power consumption (Figure 4(b)). It is worth mentioning that this feature has rarely been found in other materials. Subsequently, the author analyzed the energy bands of CdSe QDs and SF, and found that there were many hole-trapping centers in the interface energy gap, which proved that the unipolar charge transport of holes could be the main reason for the device to display multi-level capabilities. Combined with the device’s high endurance, Rabinal’s work provided reference value for the next generation of multi-bit RRAM.

Carbon quantum dots (CDs) and semiconductor QDs have good optical stability and similar properties. Based on this characteristic, Lv et al. prepared an optically adjustable resistance memory of CDs doped silk protein as a dielectric layer in 2018, and studied the mechanisms using different top electrodes (Al, Au and Ag) (Figure 4(c)). Both Al and Au-based devices exhibited WORM characteristics under dark and light conditions, but Ag-based devices exhibited stable two-stage data storage under illumination (Figure 4(d)). Through various characterization methods, the authors concluded that space charge limited conduction (SCLC) is the mechanism of Al and Au-based devices. Simultaneously, through the scanning electron microscopy (SEM) analysis of the Ag/CDs-silk/Ag planar device, it was proved that the formation of Ag CF can explain the RS mechanism of the device (Figure 4(e)). Furthermore, the authors also demonstrated that the device’s set voltage is decreasing due to the synergy between photovoltaic and optical gate effect owing to the enhanced internal electric field. In the same year, Srinivasan et al. discovered the bistable memory characteristics based on CdTe QDs-doped gelatin as the active layer [55]. Subsequently, RRAM based on QDs doping RNA was reported, which further indicated that biomaterials doped with QDs have great potential in the development of new generation of electronic devices [56].

#### 2.3.2. RRAM based on 2D materials-doped biomaterials

In addition to common hybrids of QDs and biomaterials described above, there are also some hybrids of two-dimensional (2D) materials and biomaterials for the application of RRAM, such as molybdenum disulfide nanoparticles (MoS_2_), graphene oxide (GO), etc. Being an excellent semiconductor material, MoS_2_ has been widely used in various electronic devices for its electrical conductivity, optical properties, and biocompatibility. On the other hand, as one of the most famous biomolecules, deoxyribonucleic acid (DNA) has a wide range of applications in nanotechnology. Recent research shows that DNA biopolymers can also be widely used in electronic and optoelectronic devices, such as organic light-emitting diodes (OLEDs), transistors, memory devices, etc. Choi et al. reported the heterogeneous structure of MoS_2_ and DNA for resistive memory in 2019. Carboxyl-modified molybdenum disulfide can be fixed on DNA to form heterogeneous layer and DNA can be arranged on the electrode through sulfhydryl groups. By analyzing the electrical properties of Au electrode-based device, the author discovered that the device had excellent characteristics with small current (nA), wide voltage distribution (−4 V to 4 V) and high retention time (10 days). In the case of forward voltage bias, electrons were injected into MoS_2_ and tunneling occurred under high-voltage excitation, causing LRS. In this process, both the charge donor and acceptor are served by MoS_2_-DNA heterogeneous layer [57].

In recent years, there have been increasing scientific researches on 2D semiconductor materials, among which graphene oxide (GO) has attracted extensive attention for its high surface activity and unique carrier transfer characteristics. Initially, researchers have studied resistive switching effects in GO-doped PVA and TiO_2_ thin films. Inspired by this, researches introducing GO into biological matrix for resistance switching have also been carried out. GO-doped type-A gelatin was used as the resistive layer, Al and ITO were adopted as the top and bottom electrodes, respectively. Although no obvious resistance switching phenomenon was observed, it was a benign exploration of GO biological matrix for RRAM research. Then, in 2019, Liu et al. embedded GO into another kind of biological materials, namely carboxymethyl cellulose (CMC), which made an Al/CMC-GO/Al structure of memory on flexible PET substrate (Figure 4(f)). The device exhibited favorable WORM characteristics and could maintain high switching current ratio (10^5^), low set voltage (2.22 V) and excellent retention time (10^4^). In addition, the mechanism of HRS to LRS can be explained by the SCLC model. In conclusion, GO and CMC composites make it possible to further develop biomaterials-based flexible electronic devices [58].

### 2.4. RRAM based on hybridization of other materials and biological materials

Composites with nanowires and surfactants in biomaterials have also been reported. Among them, composite materials based on Ag nanowires and silk showed fine threshold switching characteristics in flexible RRAM devices, which can be used for sensor array addressing and electronic skin [59]. Moreover, the manufacture of DNA templates based on a variety of metal materials has a wide range of applications in biomolecular science, and this property can be combined with the existing processing of DNA biopolymers and the rapid photoinduction of metal precursors in DNA biopolymers to develop functional organic devices [60]. In 2011, Hung et al. reported that DNA was treated with cationic surfactant cetyltrimethylammonium (CTMA) to form a DNA-CTMA polymer, and then embedded with Ag NPs using photochemical reactions. The authors found that ultraviolet light can effectively adjust the properties of the composite polymer, so that the device showed excellent WORM performance. Biopolymer materials were directly compounded with metal NPs, which resulted in a functional device that can be used in DNA research [15]. Afterwards, the authors succeeded in controlling WORM and WREM (write-read-wipe memory) characteristics via changing the illumination time of biomaterial polymers (Figure 4(g)). In recent years, the authors used DNA-CTMA polymer as the resistance layer to study the device characteristics under different top electrode conditions (Cu, Ag, Al). When the device structure was Ag/DNA-CTMA/ITO, it showed the characteristics of bipolar non-volatile memory, and the switching current ratio was greater than 10^2^ while the retention time was greater than 10^4^ among 200 test cycles [61 62,].

Starch is a kind of cheap, abundant, degradable and biocompatible polymer material, which has been widely studied as a solid polymer electrolyte in electrochemical system [63]. Based on successful application of polysaccharides to nanoscale electronic memory devices, Raeis-Hosseini and Lee explored the possibility of starch as a flexible biomolecular material in 2016, and a composite of starch and chitosan was used as the resistance layer. By controlling the chemical components in the starch, the behavior of the flexible device could be gradually regulated, which was expected to be used in the study of artificial synapses [64]. Then, in 2017, Liu et al. reported that WK@ AuNCs-SF-based RRAM device can be used to simulate the synapse, and WK@AuNCs can be considered as electronically charged nanoparticles (Figure 4(h)). Compared with the pure SF-based devices, WK@AuNCs-SF-based RRAM device worked under the smaller voltage and had the more stable set process (Figure 4(i)). Experiments combining WK@AuNCs-SF with mouse preosteoblast cells have strongly demonstrated that biocompatibility and synaptic capacity implied the great potential of biocomposites to mimic artificial synapses [28].

## 3. Biocomposite for devices based on transistor structure

### 3.1. FET

FET was initially put forward by J.E. Lilienfeld, who got a patent due to his idea in 1930. He proposed that a FET behaves as a capacitor with a conductive channel between source electrode and drain electrode. The applied voltage on the gate electrode controls the amount of charge carriers flowing through the conductive channel. The first FET was prepared and fabricated by Kahng and Atalla in 1960 using metal-oxide semiconductors (MOSFETs) [65–73]. In recent years, rising materials and manufacturing costs and public interest in more environmentally friendly electronic materials have supported the rapid development of organic electronics [74–76]. The emergence of the first polythiophene-based FET in 1986 has aroused widespread interest in organic field effect transistors (OFET). In just 30 years, this field has made rapid progress. OFET has potential and extensive applications in many fields such as OLEDs, organic light detectors, organic solar cells, sensors, organic data storage devices, and flexible flat panel displays due to its large-area fabrication ability [2].

A typical OFET mainly consist of two structures: bottom-gate and top-gate. Further, bottom-gate and top-gate structures include top-contact and bottom-contact structures, respectively. The structure of OFET is similar to a capacitor, source/drain electrodes and conductive channel of organic semiconductor film act as one electrode plate, and the gate electrode is equivalent to another electrode plate. For typical p-type OFET, when a negative voltage called V_GS_ is applied to the gate electrode, positively charged holes are induced in the semiconductor layer near the insulating layer, and negatively charged electrons are accumulated at the gate. At the same time, a negative voltage named V_DS_ is applied between the source and drain electrodes, and a current I_DS_ will be generated between the source and drain electrodes. By adjusting V_GS_, the electric field strength in the insulating layer can be controlled, and the density of the induced charge is dependent on the electric field. Therefore, the width of the conductive channel between the source and the drain will be various, and the current between the source and the drain will change. Hence, the purpose of adjusting the current between the source and the drain can be obtained by adjusting the electric field strength in the insulating layer. If the V_DS_ keep unchanged, when the applied V_GS_ is small, the I_DS_ is low, which is called the ‘off’ state, when the V_GS_ is large, the I_DS_ reaches a high value, which is called the ‘on’ state. In this section, we concentrate on the development of biocomposite-based transistors and their functional application [2,18–21,73 76,].

### 3.2. Biocomposite-based flash memory

The common feature of non-volatile memory is that the stored data will be retained even when the system power is off. Flash memory has been got a lot of interest due to higher chip density, multi-bit per cell storage properties and compatibility with the current complementary metal-oxide-semiconductor (CMOS) technology. On the other hand, the manufacturing cost of flash memory is much lower than that of EEPROM because byte-erasable EEPROM requires more area than block-erasable flash memory. Therefore, flash memory becomes most important and most widely adopted technology for non-volatile solid-state storage. According to the McClean Report, the flash memory occupied more than 40% market of the MOS memory IC market. Flash-memory-based on transistor structure can be further classified into two types-floating gate flash memory and charge-trapping flash memory [74].

#### 3.2.1. Floating gate flash memory

**Figure 5.**
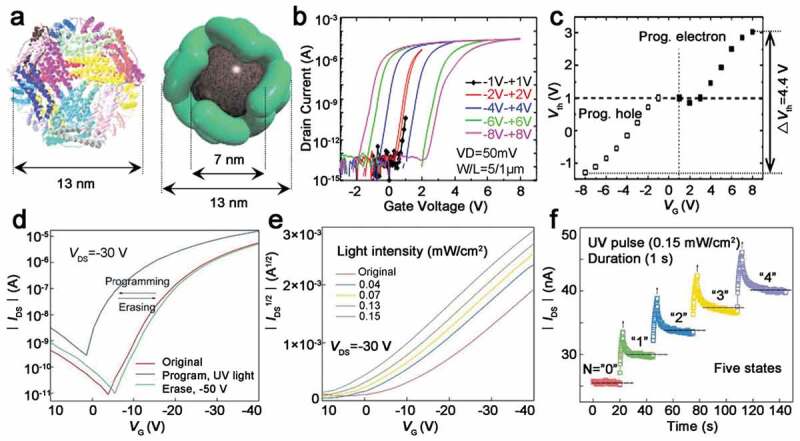
(a) Molecule structure of ferritin ribbon and ferritin/inorganic Co-BND biocomposite. (b) Transfer characteristics of ferritin/Co-BND-based floating gate flash memory with different V_G_ range. (c) V_th_ shift after programming and erasing operation with different V_G_. Reprinted with permission from [78]. Copyright 2008 AIP Publishing. (d) V_th_ shift in the transfer curves at V_ds_ = −30 V with optical programming (365 nm, 0.153 mW/cm^2^) and −50 V erasing. (e) Multilevel V_th_ of CDs/silk-based flash memories under 365 nm illumination with different intensities. (f) Five different states of photonic flash memory device obtained with five continuous UV pulses for 1 s. Reprinted with permission from [83]. Copyright 2019 WILEY‐VCH

Floating gate flash memory has the similar structure with conventional FET. The typical structure of FET contains a gate electrode, a gate dielectric whose function is to adjust charge carriers of conductive channel, a semiconductor layer, a source electrode and a drain electrode. For transistor type floating gate flash memory, a floating gate layer is inserted between the semiconductor layer and gate dielectric. By applying the gate bias VGS, floating gate layer can trap/de-trap the charge carriers from conductive channel. Nanostructure materials, biomaterials, organic small molecules, 2D materials and their composites are the frequently used materials employed as a floating gate [2]. Biomolecules are widely used in the construction of electronic devices because of their uniform structure, molecular recognition and self-assembly [77]. In addition, some proteins and DNA can recognize inorganic materials, such as nanoparticles and nanowires, and combine with them to form functional biocomposites [78 79,]. This function is known as biomineralization. Strict uniformity of biomolecules results in the structural uniformity of biomineralized inorganic nanomaterials and the self-assembly property of biomolecules enable the formation of ordered nanomaterials. Based on this, in 2008, Miura et al. reported a floating gate memory device utilizing uniformly sized cobalt oxide (Co_3_O_4_) bionanodot (Co-BND) architecture assembled by a cage-shaped supramolecular protein template as floating gate materials (Figure 5(a)) [78]. The biochemically synthetic ferritin/inorganic Co-BND hybrid nanodot can form a well-ordered, high-density, uniform and discrete node array. The aerial density of node array was more than 6.5 × 10^11^ cm-^2^, which was beneficial to the performance of floating gate memories. The transfer characteristics and V_th_ shifts of ferritin/Co-BND-nanocomposite-based flash memory are presented in Figure 5(b,c). The applied gate voltages were increased from ±1 V to ±8 V. When a small gate voltage of ±1 V was applied, the memory window was almost 0 V. The memory window increased from 1 V to 4.4 V with the scan voltage increased from ±1 V to ±8 V. Meanwhile, compared to the ferritin/Co-BND-nanocomposite-based flash memory, the transistor without floating gate layer exhibited no memory window. The result indicated that no carriers were injected into the floating gate layer when the scan gate voltage was small, and the carriers could be injected and confined in the ferritin/Co-BND floating gate layer when scan gate voltage was large enough. In conclusion, the prepared flash memory showed non-volatile data storage and nice device properties characteristic due to the effective charge-trapping feature of Co-BND. In 2014, the authors reported a flexible and transparent nanocrystal floating gate memory device using carbon nanotube-CdS nanostructures embedded in silk protein matrix again [80]. CdS-decorated multi-wall carbon nanotubes (MWCNTs) were dissolved in silk solution to prepare silk-based flash memory devices. The ITO/MWCNT-CdS&silk biocomposite/Al memory device exhibited a large flat-band voltage drift memory characteristic because holes were trapped in MWCNT-CdS&silk biocomposite. With the increasing of scan voltage, an increase of the memory window was observed.

Due to the rapid development of big data and limit of Moore’s law, electronic devices are trending towards miniaturization. Photonic transistor, which can be used as light sensing and data storage device, is an important research field in order to adapt to this trend. Photonic transistor can be regulated by light and electricity at the same time because of introduction of photoactive materials. Biocomposite with biomaterial and photoactive material is a promising candidate to work as building block of photonic transistor to reduce the cost of device preparation [69,81].

In 2012, Chen et al. showed a protein transistor made of an antibody molecule and two gold nanoparticles [82]. The transistor was made responsive to light with different wavelengths by attaching a photoactive quantum dot to the antibody molecule. The light-tunable capacity of protein transistor may be caused by the separation of photo-generated carriers in nanocrystals, and then the photo-generated carriers are injected into the conductive layer of the protein transistor to increase drain current. This method provides a way to fabricate versatile, miniaturized, large-scale integrable bioelectronic circuits. Lv et al. reported a photonic transistor using a blend of CDs and silk protein as photo-tunable charge-trapping layer in 2019 [83]. The biocomposite charge-trapping layer displayed stable light-induced and current-triggered charge trapping and charge releasing behavior by employing silk as stabilizing building block and CDs as photoactive element. Benefit from the introduction of photoactive CDs, the transistor exhibited optical programming and electrical erasing capacity and multi-level data storage ability. As depicted in Figure 5(d), the remarkable V_th_ shift was obtained after an application of UV light with the wavelength of 365 nm and intensity of 0.153 mW/cm^2^ as programming operation. When the UV programming carried out, the holes transferred from CDs-silk charge-trapping layer to pentacene layer and accumulated in the pentacene layer. Hence, the flash memory achieved a high conductivity and a positive V_th_ shift. On the contrary, the flash memory device can be erased to low-conductivity initial state by applying a negative gate bias of −50 V. The holes trapped in the pentacene layer were released to CDs-silk charge-trapping layer to result in a negative V_th_ shift. The difference in V_th_ between the high-conductivity state and low-conductivity state ($\Delta V_{th}=V_{th,erase}-V_{th,program}$), defined as memory window, was about 15.2 V. Further, Figure 5(e) indicates that CDs-silk-based photonic memory can realize multistage optical storage. The memory device initiated at state ‘0’. Then, 4 UV pulses for 1 s were followed in order to program the memory device to ‘1’, ‘2’, ‘3’, ‘4’ states, respectively (Figure 5(f)). Because the volatile and non-volatile states are switchable in altered illumination conditions, short-term plasticity (STP) and long-term plasticity (LTP) behaviors of synapse were mimicked by the prepared single transistor.

#### 3.2.2. Charge-trapping flash memory

**Figure 6.**
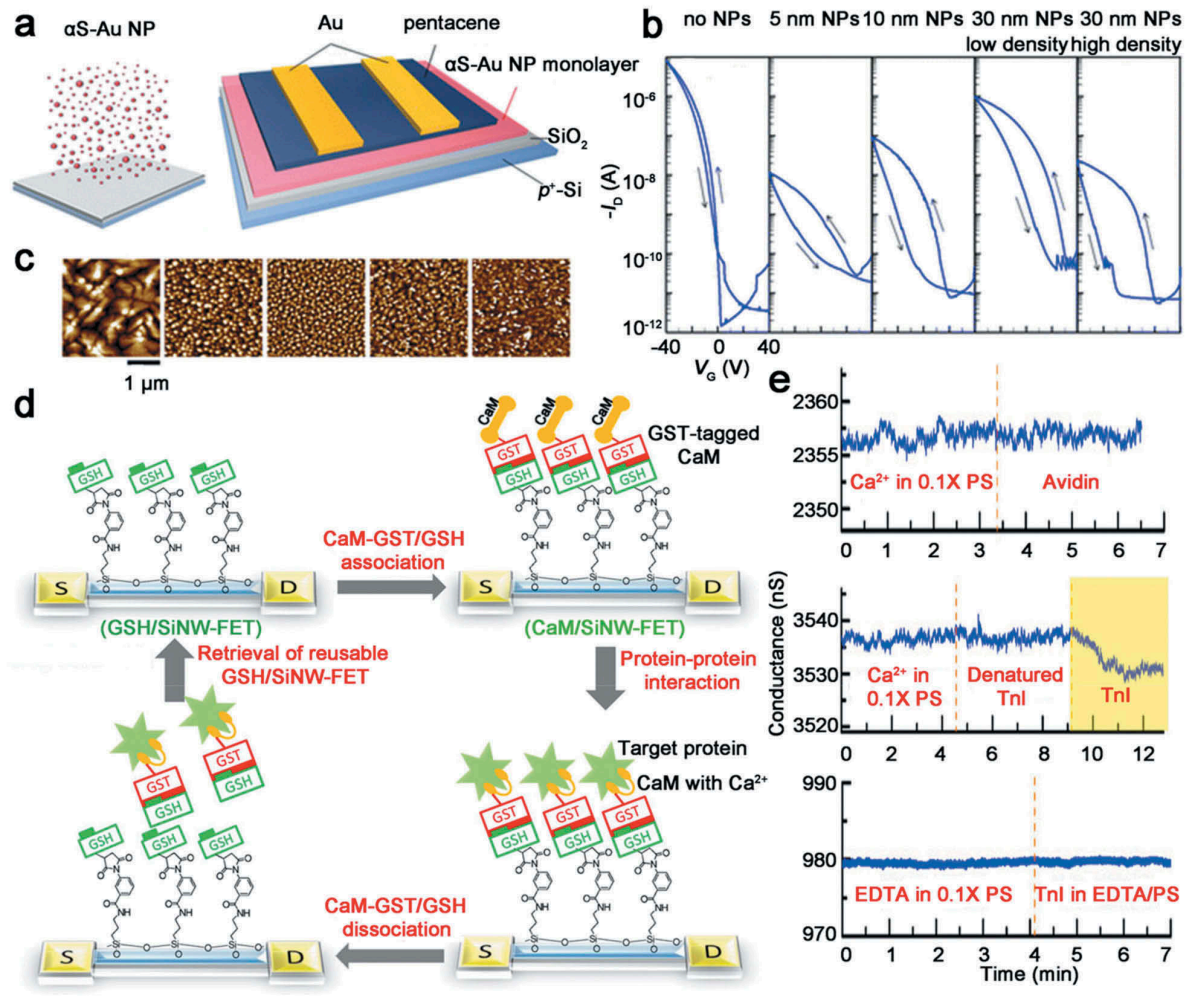
(a) Sketch representation of synthetic αS-Au NPs biocomposite and structure of αS-Au NPs biocomposite-based charge-trapping flash memory device. (b) Transfer characteristics of flash memory with Au NPs of different size and distribution as charge-trapping sites. (c) AFM topographic images of 50-nm thick pentacene semiconductor layer corresponding to (b). Reprinted with permission from [84]. Copyright 2016 American Chemical Society. (d) Preparation strategy of reusable calmodulin-modified silicon nanowire field-effect transistor biosensor. (e) Control trials to examine the specificity of CaM-binding proteins biosensor. Reprinted with permission from [95]. Copyright 2010 National Academy of Sciences

Charge-trapping flash memory has the similar structure as floating gate flash memory. The charge-trapping flash memory is composed with a gate electrode, a gate dielectric, a charge-trapping layer, a semiconductor layer, source and drain electrodes. The only difference between the charge-trapping flash memory and floating gate flash memory is that the charge-trapping layer is insulator materials with electret property, whereas floating gate layer is usually conductor. Biomaterials and their nanocomposites have huge application potential for eco-friendly charge-trapping layer materials due to their biodegradability and charge storage ability [2 69,]. In 2016, Kim and co-workers demonstrated an OFET charge-trapping flash memory with highly controllable memory performance using alpha-synuclein-Au nanoparticles (αS-Au NPs) biocomposite (Figure 6(a)) [84]. Although enormous potential of nanoparticle-based organic transistor memories had been demonstrated, device fabrication processes are still desired to be improved. For example, nanoparticles aggregations are formed by random diffusion kinetics, so it is difficult to precisely control their size and distribution. αS can bind with Au NPs due to their disorder structure and strong binding affinity, and the αS-Au NPs can strongly bind to SiO_2_ to form a tightly distributed charge-trapping layer by simple solution deposition, which has a uniform distribution and a controllable size [84 85,]. The transfer curves and corresponding AFM topographic images of 50-nm thick pentacene semiconductor layer of prepared charge-trapping flash memory devices are presented in Figure 6(b,c). The memory window of nanoparticles free transistor is almost 0 V. Contrarily, the nanoparticles containing flash memory transistor exhibited increased memory window. The result demonstrated that the αS-Au NPs composite served as carriers trapping center. During the sweeping operation, the nanoparticles can function as charge trapping and de-trapping layer. Therefore, the memory device with Au NPs showed an increscent memory window. In addition, memory performance of αS-Au NPs was affected by the size and density of Au NPs. By controlling the particle size and distribution, the authors optimized the αS-Au NPs based transistor memory performance, including the current level, hysteresis window and retention time. For small-sized nanoparticles, memory devices possessed the short-term memory and fast writing/erasing ability, which was appropriate for neuromorphic computing application. In contrast, large and dense nanoparticles were the excellent choice for charge-trapping layer of non-volatile charge-trapping flash memory due to their strong charge-trapping ability and long retention time.

We do not aim to present all related works in this review. We merely compare the performance of some recently reported biocomposite-based transistor flash memories in Table 2. Despite the fact that significant progress has been made, more research is needed to increase the mobility and enhance the stability of transistor-based flash memory.

**Table 2.** A performance comparison of biocomposite-based flash memory

Biocomposite unit	Window (operation voltage)	Channel length/width (μm)	ON/OFF ratio	Endurance	Retention (s)	Mobility (cm^2^/Vs)	Ref
Ferritin/Co-BND	4 (±10)	–	–	10^5^	10^4^	200	[78]
Silk/MWCNT/CdS	9.8 (±10)	–	–	–	10^3^	–	[80]
Silk/PVA	5	100/10,000	10^4^	300	10^4^	0.22	[86]
Silk/CDs	16.8 (±50)	50/1000	10^5^	500	7 × 10^6^	–	[83]
α-synuclein/AuNP	40 (±60)	50/500	10^4^	7	10^7^	–	[84]
Maltoheptaose-b-PS	52.7 (±50)	50/1000	10^8^	200	10^4^	0.59	[87]
Chitosan/Y_2_O_3_	–	–	10^5^	–	–	0.09	[91]
Chitosan/H_3_PO_4_	40 (±60)	50/500	10^6^	7	10^7^	6.36	[92]
DNA-CTMA	7 (±10)	35/3000	10^4^	2	10^3^	–	[73]

### 3.3. Biocomposite-based biosensor

Small organic molecules and polymers based OFET sensor have been successfully employed to detect various analytes in vapor, ion and optical systems. To meet the market demand of environmental monitoring, food safety, and preventive medical examinations, biomolecule-based biosonsers are expected to be widely used to detect a variety of chemical analytes. A field effect transistor biosensor is a sensor based on the structure and working principle of FET [88–90]. It replaces the metal gate in the FET structure with an ion-sensitive membrane or an ion-sensitive membrane modified by a biomolecule (antibody, antigen or DNA, etc.) [76,93–95]. When charged biomolecules recognize and form complexes on ion-sensitive membranes, or biomolecules undergo biochemical reactions on ion-sensitive membranes to form ionic products (such as H^+^), the potential changing of the ion-sensitive membrane is equivalent to adjusting the gate voltage by an external power source to achieve the purpose of controlling the channel current between the source and the drain. Moreover, the adsorption amount of biomolecules on the gate-sensitive film has a linear correlation with the drain output current within a certain range. Therefore, by measuring the magnitude of the drain current, it is possible to quantitatively analyze the biological reactions that occur on the gate ion-sensitive membrane [96]. These biological reactions include nucleic acid hybridization, protein interaction, antibody-antigen binding, and enzyme substrate reactions.

In 2010, a label-free detection of protein–protein interactions using a calmodulin (CaM)-modified nanowire transistor biosensor was showed by Lin et al. [95]. CaM is an acidic protein that regulates the binding of calcium ions. CaM that binds to calcium ions can combine with different proteins to regulate the physiological activities of the human body. The authors designed a CaM-modified silicon nanowire field-effect transistor (SiNW/FET) to detect calcium ions and related proteins. The reversible binding of CaM to the surface of SiNW-FET is based on the combination of glutathione (GSH) and glutathione S-transferase (GST). The authors developed a CaM/SiNW-FET by utilizing the reversible binding and dissociation between GSH and GST-labeled CaM (CaM-GST). GSH combines with SiNW-FET to form GSH/SiNW-FET, and then CaM-GST combines with GSH to form CaM/SiNW-FET to detect various target proteins through CaM–protein interaction. When the CaM and SiNW-FET are dissociated, the device went back to its original state (GSH/SiNW-FET). This device preparation strategy turns sensitive nanowire transistors into reusable biosensors for rapid screening of potential CaM-binding proteins (Figure 6(d)). Troponin can bind to CaM under calcium conditions and is used as a marker for the diagnosis of myocardial infarction. The authors carried out the detection of cardiac troponin I (TnI) using the designed CaM/SiNW-FET in the condition of calcium ions. After TnI was added, the conductance was decreased. The decrease of conductance in CaM/SiNW-FET channel was caused by the positive charge of TnI carried at pH 7.4. In order to examine the specificity of this effect, the authors carried out several control trials. The top graph presented in Figure 6(e) indicates that CaM/SiNW-FET shows no conductance change to avidin, which is also a positively charged protein. In addition, as shown in the middle panel of Figure 6(e), the biosensor did not respond to degenerative TnI, but the same device could detect TnI as is shown in the yellow region of Figure 6(e). Finally, in the absence of calcium, the device did not respond to TnI (Figure 6(e), bottom panel).

In addition to protein, DNA aptamer with the advantages of affinity with graphene, relatively short chain length, strong selectivity, and high analyte variability is an excellent graphene FET (GFET) sensor element. Single-stranded DNA was selected as the functionalized layer due to its wide range of affinity target molecules and π-π superposition to interact with graphene. Kybert et al. reported scalable arrays of transistor-type sensors based on DNA-decorated graphene using hotolithographic processing technique [76]. These devices have excellent sensor characteristics, including fast response, low electronic noise, detection limits below 1 part per million for some analytes, and the capacity to distinguish very similar analytes, especially structural isomers.

## 4. Biocomposite for artificial synaptic applications

**Figure 7.**
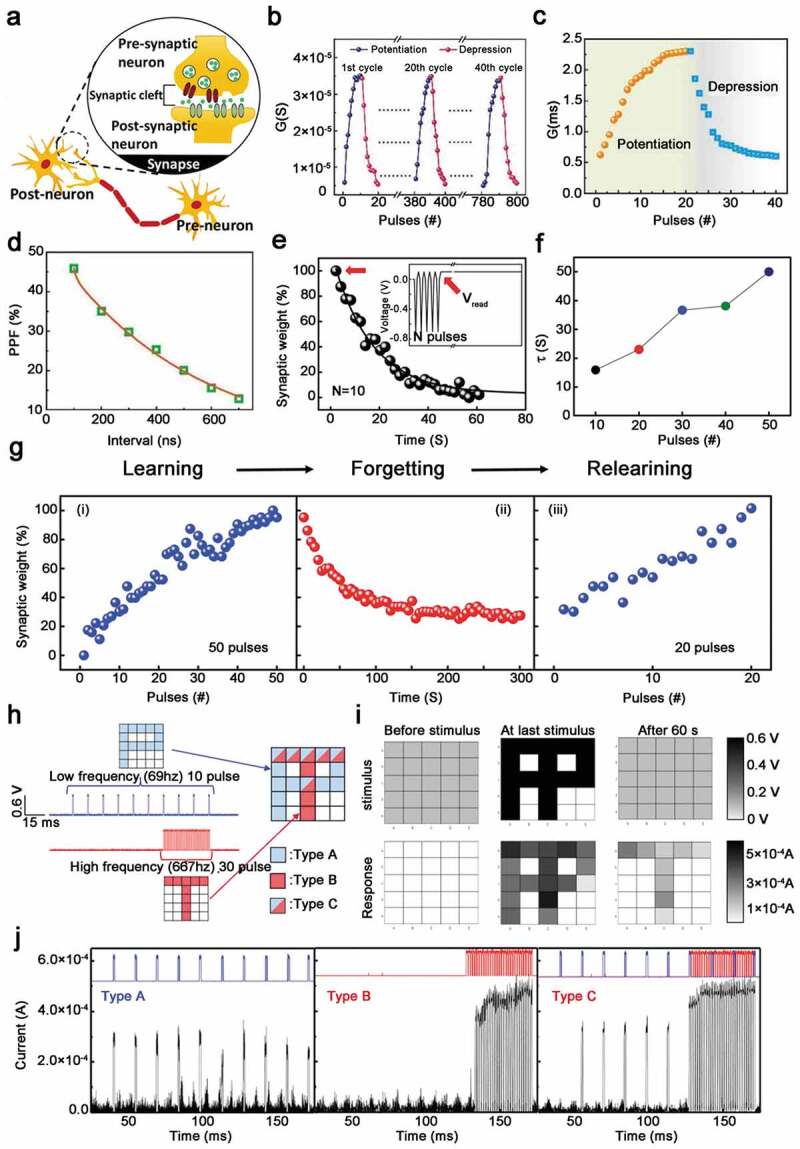
Electrical properties and applications of artificial synaptic devices based on two-terminal device.﻿ (a) Schematic diagram of synaptic plasticity regulation from pre-synaptic neurons to post-synaptic neurons. Reprinted with permission from [108]. (b, c) Repeated enhancement and suppression characteristics of AgNCs@ SF-based memristors. (d) PPF index is a function of the pulse interval time (from 100 to700 ns). (e) Changes in synaptic weight under different 10 continuous voltage pulses. Reprinted with permission from [11]. Copyright 2019 WILEY‐VCH. (f) Transition from short-term plasticity (STP) to long-term plasticity (LTP) caused by continuous stimulation. (g) The conductance is enhanced under 50 consecutive pulse sequences, simulating the learning function of the human brain. Synaptic weight decays with time for 300 s, simulating the forgetful process of the human brain. Subsequently, the re-learning function of the human brain is simulated, and 20 consecutive spikes are applied to increase the synaptic weight to the state after the first learning process. Reprinted with permission from [35]. Copyright 2017 American Chemical Society. (h) Use biocompatible artificial synaptic array to memorize two pictures, input 10 times of low-frequency P and 30 times of high-frequency T on a 5 × 5 biocompatible artificial synaptic array to memorize the letters of P and T. (i) Biocompatible artificial synaptic array applied stimulation and current response before stimulation, at the last stimulation and after 60 s. (j) The current response of an artificial synapse of three stimuli types as shown in (h). Reprinted with permission from [108]. Copyright 2018 American Chemical Society

Neuromorphic computing is an attractive research area, which will further overcome the von-Neumann bottleneck in the limited data transmission speed of memory and information processing. In order to simulate the synaptic plasticity of the biological nervous system, ultra-small-sized synaptic electronics are emphasized as a basic component of neuromorphic computing systems [97–100]. In addition to data storage devices based on bio-oil cubes, another attractive area of research is studying biological memory systems, and then simulating the structure and memory functions of these systems. In particular, the neural neuromorphic network of spikes consists of an array of interconnected pre-neurons and post-neurons, and synaptic plasticity triggered by chemical transmission is related to the temporal diversity of post-neurons and pre-neurons (Figure 7(a)). Typically, a chemical synapse, consisting of axons, synaptic spaces, and dendrites, is considered a functional connector that allows neurons to pass neurotransmitters to neighboring neurons. Synaptic plasticity and memory (SPM) hypothesis hold that synaptic plasticity is a straightforward decisive factor for acquiring memory and learning ability, synaptic plasticity also refers to increasing or decreasing of synaptic neural activity when it changes. According to the duration, synaptic plasticity can be divided into short-term plasticity (short-term enhancement and short-term inhibition) and long-term plasticity (long-term enhancement and long-term inhibition). In particular, long-term enhancement (LTP) is one of the most studied forms of synaptic plasticity. Therefore, the permanent change in the strength of synaptic connections plays a vital role in the consolidation of memory and the formation of durable memory [101]. Furthermore, development of artificial synapses mimics the activity-dependent long-term potential-controlled synaptic plasticity, paving the way for advanced information exchange inter-connectivity, which will further facilitate the development of highly integrated and low-power memory devices. The main feature of artificial synaptic materials is the realization of functions, including multi-level data storage and indispensable non-volatile properties [102–104]. Biological synaptic systems can establish a link between their response and the history of input stimuli, which should act as a multi-state memory and respond to the same repeated external input stimuli. On the other hand, in order to avoid refreshing or energy dissipation of the memory state during data storage, the non-volatile nature of the memory elements is essential [105]. So far, based on the existing device structures, including two-terminal and three-terminal devices, we have summarized the latest artificial synapses in this research area.

### 4.1. Biocomposite for two-terminal synaptic device

Biologically, neural memory is regarded to be closely related to synaptic weight and the strength of synaptic connections. In 2019, Liu and co-workers demonstrated new mesoscopic bioelectronic hybrid materials of silk fibroin (SF)-Ag nanoclusters (AgNCs@BSA; BSA: bovine serum albumin) based two terminal memristor-based synaptic devices [11]. Using AgNCs@BSA as an electron potential well enhanced the transport behavior of electricity in the SF film, which made the switching speed of the SF memory resistor significantly increased, that also showed unique synaptic performance and synaptic learning ability. In the connection between neuron synapses, the concentration of ionic species limited the activation/inhibition of the release of neurotransmitters and receptors for a certain period of time, thereby controlling the increase and decrease of synaptic weights (Figure 7(b)). The authors made use of the electrical conductivity of the two devices as a synaptic weight, and obtained a behavior similar to the nonlinear transmission characteristics of biological synapses. In addition, the author applied 250 ns width and 1.5 V positive and negative pulses to the device, and obtained the response of the change in synaptic weight. As the positive pulses increased, the conductance was enhanced, conversely, by changing the polarity of the pulses to the opposite, the conductance of the device decreases as the number of pulses increase (Figure 7(c)). In biological synapses, the synaptic weight increases as the interval between stimuli decreases, and this change is due to the increase in presynaptic Ca^2+^ concentration leading to the release of synaptic neurotransmission, what is more, this behavior is similar to that of PPF in biological synapses [106]. PPF is defined as

(1) $$PPF=\frac{G2-G1}{G1}*100\%$$

where G1 and G2 are the conductance reading after the first and second pulses, respectively. Therefore, the time constants τ1 (10 ns) and τ2 (509 ns) of these two accessories correspond to fast and slow decay conditions, respectively (Figure 7(d)). These time scales are consistent with biological synapses.

Generally, synaptic plasticity can be divided into long-term plasticity (STP) and short-term plasticity (LTP) based on synaptic retention time [107]. Lee and Park made a flexible lignin artificial synaptic device for simulating several basic synaptic behaviors, including simulated memory conversion, STP, LTP, plasticity that depends on spike rates, and short-to-long-term conversion in 2017 (Figure 7(e)) [35]. In order to measure the conversion from LTP to STP, Lee applied different numbers of pulses as stimuli before the synapse, utilizing a continuous voltage bias (0.1 V) to read the change in current (Figure 7(f)). Firstly the current fell rapidly, then gradually decreased and finally reached an intermediate level. Interestingly, 20 pulses were enough to obtain the same synaptic weight before spontaneous decay occurred, a phenomenon analogous to the brain’s forget-memory feature, which could memorize previously forgotten information faster than the first time, and the figure illustrates it in detail (Figure 7(g)) [35]. In 2018, Lee demonstrated the use of biomaterial and Ag dynamics to simulate synaptic functions. This study confirms that it is possible for ι-Carrageenan (ι-car) to build artificial neuromorphic systems using biocompatible synapses (Figure 7(h)) [108]. He used a 5 × 5 array to stimulus, the device was in a closed state, and the synapses showed low electrical conductivity. To our knowledge, electrical stimulation can cause the formation of silver conductive filaments and change the resistance state. The application of low-frequency stimulation temporarily increased the conductance of the device and caused the formation of weak conductive filaments. Therefore, the image of ‘P’ was only temporarily stored in the array and would be forgotten quickly. However, when high-frequency stimulation was used, the synaptic weight of the device was increased and thick conductive filament could be formed, so only the images of ‘T’ were stored in the synapses array after 60 s. Under a weak stimulus, the increase in the conductance of the A-type pixel was limited, and the synapse returned to an insulated state. However, B-type and C-type pixels can maintain high conductance for a prolonged period of time under strong stimulation, because strong stimulation amplified the increase in conductance and induced the formation of strong filaments (Figure 7(j)) [108]. By inducing the formation of stable conductive filaments, repeated stimulation caused the transition from STP to LTP. Furthermore, this transformation is similar to the brain’s memory process.

### 4.2. Biocomposite for three-terminal synaptic device

There are two limitations to the development of synaptic devices based on two-terminal devices. One is the delayed transmission of signals, and the other is the transmission of signals through a single channel between the post-neuron and pre-neuron spikes. Synapses based on three-terminal FET can transmit signals through the channel medium, and at the same time realize the learning events through the control of neurons.

**Figure 8.**
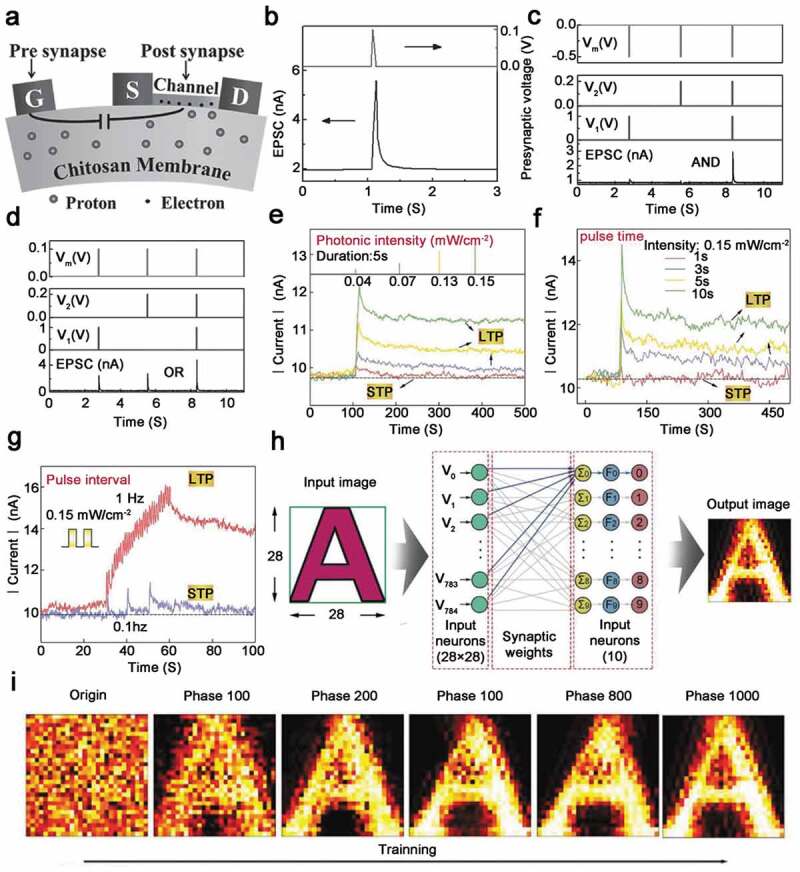
Application of field effect transistor in synapse. (a) Schematic diagram of a synaptic transistor. (b) EPSC is triggered by presynaptic pulses (0.1 V, 10 ms) and constant V_ds_ (70 mV). (c) The positive spikes applied to the controlled gate are tuned to implement logic ‘AND’ functions. (d) Realize ‘OR’ logic function by adjusting the input and output characteristics by applying negative spikes on the controlled gate. The formation of STP or LTP in photon synapses depends on the input light pulse intensity. Reprinted with permission from [109]. Copyright 2015 WILEY‐VCH. (e) Light pulse duration (f) and pulse interval (g). (h) The system-level MINIST mode simulation based on the light-response transistor, 785 input neurons and 10 output neurons constitute the SLP network. (i) The input image A is output, and as the training is strengthened, the mapped image A is output. Reprinted with permission from [83]. Copyright 2019 WILEY‐VCH

In 2015, Wan et al. prepared an independent artificial synapse based on oxygen crystals on the chitosan membrane (Figure 8(a)) [109]. Under repeated high pulsed voltage, proton-related electrochemical doping occurred in the indium zinc oxide (IZO) channel layer, which caused the transition from short-term memory to long-term memory. When a positive synapse was applied on the gate, protons were driven laterally and accumulated at the chitosan/IZO channel interface. Owing to proton/electron electrostatic coupling, electrons accumulated and leakage current increased. When a pre-synaptic potential (0.1 V, 10 ms) was applied to the gate, and a voltage of 70 mV was applied between the source and drain for excitatory postsynaptic current (EPSC) measurement, EPSC reached a peak of 5.6 nA and gradually decayed to the initial value of 1.9 nA (Figure 8(b)). This EPSC behavior was very similar to the EPSC process in biological excitatory synapses. Interestingly, they also made pulse logic operations by this artificial synapse. When pulses were applied to one or two pre-synaptic inputs, post-synaptic current could be measured, and the positive and negative pulses on the control gate were used to adjust the logic function ‘OR’ or ‘AND’ (Figure 8(c,d)) [109].

In addition, photonic synapses have been proven to have faster speeds, higher bandwidth, lower energy consumption, and less light-stimulated crosstalk. Lv et al. achieved STP and LTP in optical synaptic device based on carbon dots/silk protein (CDs/silk), and enhanced simulation of STP and LTP was realized by illumination with different intensities (Figure 8(e)) [83]. When a pulse with a lower light intensity was introduced, the device cannot maintain a high conduction state, but decayed back to the initial state over time, similar to biological STP mechanism. In contrast, when the intensity of the input optical signal was increased, the optical synapse also permanently transferred to a high conduction state, simulating LTP behavior (Figure 8(f)). Similarly, the realization of STP and LTP was also achieved by operating artificial synapses at key pulse duration or repetitive intervals (Figure 8(g)). Furthermore, they built a CDs/silk-based synaptic device to simulate a supervised learning framework and execute a system-level simulation of MINIST pattern recognition, 784 synaptic weights in the mapped image connected to the output letter ‘A’ are described as a function of the learning time (Figure 8(h)). Compared with the original chaotic mapping image, the rearranged mapping image after 100 learning stages presented a recognizable shape of the learning letter ‘A’, and as the number of learning stages increases, the learning letter ‘A’ becomes thicker, and the learning letter ‘A’ reduced white space in the shape. As the training progressed, the recognition accuracy gradually became saturated with small fluctuations (Figure 8(i)). In the SLP network, after 15,000 learning stages of ‘A’ and ‘I’, the recognition accuracy rates reached 73% and 65%, respectively [83]. Synaptic transistors based on hybrid biological materials provide a promising way for biologically inspired neuromorphic computing [110].

## 5. Conclusions and perspective

The employment of fascinating natural materials in organic electronics opens up new avenues for studying sustainability, biodegradability, and biocompatibility devices, and has been applied in various fields. It is necessary to search these unusual materials before realizing a wide range of applications. In addition, metal and semiconductor nanoparticles provide novel performance and functions for a new generation of materials and devices due to their unique electronic, optical, catalytic, and self-assembly properties. Due to the issues of size and distribution of nanoparticles, it is often necessary to stabilize them with functional monolayers, polymer films, and natural biomaterials. Therefore, biocomposites provide an excellent choice for the future application of organic electronics. Biocomposites, especially the combination of nanoparticles and biomaterials, are very attractive research projects since it may provide new ideas for the field of nanobiotechnology. Biocomposites that combine the unique properties of nanoparticles and biomaterials provide a new type of material for physicists, chemists, biologists, and materials scientists. Several basic advantages of the biomaterial-nanoparticle hybrid system make these materials extremely attractive for future bioelectronics applications: First, the biomaterial-functionalized nanoparticles are water-soluble and have a high specific surface area. Second, the unique optical and electrical properties of nanoparticles can be adjusted and controlled by the size of the nanoparticles, and various properties of bioelectronic devices can be controlled and optimized by controlling the properties of the nanoparticles. Finally, biocomposites have good sustainability, biodegradability and biocompatibility.

The biocomposite material system has been applied in different fields, including resistive memory, flash memory, nano-biotechnology, biosensors, biofuel cells, artificial organs and artificial synapses, artificial neural network simulation, and so on. Coupling biomaterials and other specific materials with outstanding photoelectric properties, such as nanoparticles (or nanowires), into hybrid systems can generate new mutant materials and add new prospects for the development of material science and organic electronic device. In the field of bioelectronics, evolution has optimized the fascinating macromolecular structure, showing unique specific binding, replication, catalysis, and self-assembly characteristics. We believe that functional devices made with these fascinating materials will become promising candidates for commercial applications in the near future with the development of materials science and advances in device manufacturing and optimization technology. Finally, it is worth mentioning that the overall economic value of these materials will be significantly improved, which may result in a vast cost saving. At the same time, the biodegradability and bioabsorption capacity of renewable biopolymer materials provide ideas for implantable electronics. From the perspective of biomass materials to electronics, it has been reported in many fields, including microelectronic devices and biocompatible batteries based on biocomposite materials [111 112,], proving the potential of multidisciplinary integration in this research area.

## References

[1] Yang JJ, Strukov DB, Stewart DR. Memristive devices for computing. Nat Nanotechnol. 2013;8(1):13–24. 23269430 10.1038/nnano.2012.240

[2] Han S-T, Zhou Y, Roy VAL. Towards the development of flexible non-volatile memories. Adv Mater. 2013;25(38):5425–5449. 24038631 10.1002/adma.201301361

[3] Irimia-Vladu M. “Green” electronics: biodegradable and biocompatible materials and devices for sustainable future. Chem Soc Rev. 2014;43(2):588–610. 24121237 10.1039/c3cs60235d

[4] Zhou Y, Han S-T, Yan Y, et al. Ultra-flexible nonvolatile memory based on donor-acceptor diketopyrrolopyrrole polymer blends. Sci Rep. 2015;5(1):10683. 26029856 10.1038/srep10683 PMC4450595

[5] van de Burgt Y, Lubberman E, Fuller EJ, et al. A non-volatile organic electrochemical device as a low-voltage artificial synapse for neuromorphic computing. Nat Mater. 2017;16(4):414–418. 28218920 10.1038/nmat4856

[6] Lee J-S, Cho J, Lee C, et al. Layer-by-layer assembled charge-trap memory devices with adjustable electronic properties. Nat Nanotechnol. 2007;2(12):790–795. 18654433 10.1038/nnano.2007.380

[7] Han S-T, Zhou Y, Chen B, et al. Hybrid flexible resistive random access memory-gated transistor for novel nonvolatile data storage. Small. 2016;12(3):390–396. 26578160 10.1002/smll.201502243

[8] Kim S, Choi S, Lu W. Comprehensive physical model of dynamic resistive switching in an oxide memristor. ACS Nano. 2014;8(3):2369–2376. 24571386 10.1021/nn405827t

[9] Lv Z, Zhou Y, Han S-T, et al. From biomaterial-based data storage to bio-inspired artificial synapse. Mater Today. 2018;21(5):537–552.

[10] Kim S, Choi B, Lim M, et al. Pattern recognition using carbon nanotube synaptic transistors with an adjustable weight update protocol. ACS Nano. 2017;11(3):2814–2822. 28221756 10.1021/acsnano.6b07894

[11] Shi C, Wang J, Sushko ML, et al. Silk flexible electronics: from bombyx mori silk Ag nanoclusters hybrid materials to mesoscopic memristors and synaptic emulators. Adv Funct Mater. 2019;29(42):1904777.

[12] van Hest JCM, Tirrell DA. Protein-based materials, toward a new level of structural control. Chem Commun. 2001;19:1897–1904. 10.1039/b105185g 12240211

[13] Sun B, Wei L, Li H, et al. The DNA strand assisted conductive filament mechanism for improved resistive switching memory [10.1039/C5TC02732B]. J Mater Chem C. 2015;3(46):12149–12155.

[14] Lee T, Yagati AK, Pi F, et al. Construction of RNA–quantum dot chimera for Nanoscale resistive biomemory application. ACS Nano. 2015;9(7):6675–6682. 26135474 10.1021/acsnano.5b03269 PMC4642448

[15] Hung Y-C, Hsu W-T, Lin T-Y, et al. Photoinduced write-once read-many-times memory device based on DNA biopolymer nanocomposite. App Phys Lett. 2011;99(25):253301.

[16] Tseng RJ, Tsai C, Ma L, et al. Digital memory device based on tobacco mosaic virus conjugated with nanoparticles. Nat Nanotechnol. 2006;1(1):72–77. 18654145 10.1038/nnano.2006.55

[17] Nagashima K, Koga H, Celano U, et al. Cellulose nanofiber paper as an ultra flexible nonvolatile memory. Sci Rep. 2014;4(1):5532. 24985164 10.1038/srep05532 PMC4078308

[18] Kim BJ, Ko Y, Cho JH, et al. Organic field-effect transistor memory devices using discrete ferritin nanoparticle-based gate dielectrics. Small. 2013;9(22):3784–3791. 23666682 10.1002/smll.201300522

[19] Chiu Y-C, Sun H-S, Lee W-Y, et al. Oligosaccharide carbohydrate dielectrics toward high-performance non-volatile transistor memory devices. Adv Mater. 2015;27(40):6257–6264. 26332569 10.1002/adma.201502088

[20] Ko Y, Kim Y, Baek H, et al. Electrically bistable properties of layer-by-layer assembled multilayers based on protein nanoparticles. ACS Nano. 2011;5(12):9918–9926. 22092235 10.1021/nn2036939

[21] Zhang C, Shang J, Xue W, et al. Convertible resistive switching characteristics between memory switching and threshold switching in a single ferritin-based memristor. Chem Commun. 2016;52(26):4828–4831. 10.1039/c6cc00989a 26967024

[22] Jiang J, Bai ZL, Chen ZH, et al. Temporary formation of highly conducting domain walls for non-destructive read-out of ferroelectric domain-wall resistance switching memories. Nat Mater. 2018;17(1):49–56. 29180776 10.1038/nmat5028

[23] Zhao X, Fan Z, Xu H, et al. Reversible alternation between bipolar and unipolar resistive switching in Ag/MoS2/Au structure for multilevel flexible memory. J Mater Chem C. 2018;6(27):7195–7200.

[24] Moors M, Adepalli KK, Lu Q, et al. Resistive switching mechanisms on TaO_x_ and SrRuO_3_ thin-film surfaces probed by scanning tunneling microscopy. ACS Nano. 2016;10(1):1481–1492. 26738414 10.1021/acsnano.5b07020

[25] Wang Y, Lv Z, Liao Q, et al. Synergies of electrochemical metallization and valance change in all-inorganic perovskite quantum qots for resistive switching. Adv Mater. 2018;30(28):1800327. 10.1002/adma.201800327 29782667

[26] Rao F, Ding K, Zhou Y, et al. Reducing the stochasticity of crystal nucleation to enable subnanosecond memory writing. Science. 2017;358(6369):1423. 29123020 10.1126/science.aao3212

[27] Lü W, Li C, Zheng L, et al. Multi-nonvolatile state resistive switching arising from ferroelectricity and oxygen vacancy migration. Adv Mater. 2017;29(24):1606165. 10.1002/adma.201606165 28439926

[28] Xing Y, Shi C, Zhao J, et al. Mesoscopic-functionalization of silk fibroin with gold nanoclusters mediated by keratin and bioinspired silk synapse. Small. 2017;13(40):1702390. 10.1002/smll.201702390 28863240

[29] Cho D-Y, Luebben M, Wiefels S, et al. Interfacial metal–oxide interactions in resistive switching memories. ACS Appl Mater Interfaces. 2017;9(22):19287–19295. 28508634 10.1021/acsami.7b02921

[30] Lee J-W, Cho W-J. Fabrication of resistive switching memory based on solution processed PMMA-HfO_x_blended thin films. Semicond Sci Technol. 2017;32(2):025009.

[31] Gao L, Li Y, Li Q, et al. Enhanced resistive switching characteristics in Al_2_O_3_memory devices by embedded Ag nanoparticles. Nanotechnology. 2017;28(21):215201. 28462908 10.1088/1361-6528/aa6cd0

[32] Hu C, Wang Q, Bai S, et al. The effect of oxygen vacancy on switching mechanism of ZnO resistive switching memory. App Phys Lett. 2017;110(7):073501.

[33] Zhou G, Duan S, Li P, et al. Coexistence of negative differential resistance and resistive switching memory at room temperature in TiO_x_ modulated by moisture. Adv Electron Mater. 2018;4(4):1700567.

[34] Du C, Ma W, Chang T, et al. Biorealistic implementation of synaptic functions with oxide memristors through internal ionic dynamics. Adv Funct Mater. 2015;25(27):4290–4299.

[35] Park Y, Lee J-S. Artificial synapses with short- and long-term memory for spiking neural networks based on renewable materials. ACS Nano. 2017;11(9):8962–8969. 28837313 10.1021/acsnano.7b03347

[36] Gao S, Yi X, Shang J, et al. Organic and hybrid resistive switching materials and devices. Chem Soc Rev. 2019;48(6):1531–1565. 30398508 10.1039/c8cs00614h

[37] Zhu JX, Zhou WL, Wang ZQ, et al. Flexible, transferable and conformal egg albumen based resistive switching memory devices. RSC Adv. 2017;7:32114–32119.

[38] Bok CH, Woo SJ, Wu C, et al. Flexible bio-memristive devices based on chicken egg albumen: Au@SiO_2_core-shell nanoparticle nanocomposites. Sci Rep. 2017;7(1):12033. 28931861 10.1038/s41598-017-12209-6 PMC5607228

[39] Chang Y-C, Lee C-J, Wang L-W, et al. Air-stable gelatin composite memory devices on a paper substrate. Org Electron. 2019;65:77–81.

[40] Viorica GP, Musat V, Pimentel A, et al. Hybrid (Ag)ZnO/Cs/PMMA nanocomposite thin films. J Alloys Compd. 2019;803:922–933.

[41] Valentini L, Cardinali M, Fortunati E, et al. Nonvolatile memory behavior of nanocrystalline cellulose/graphene oxide composite films. App Phys Lett. 2014;105(15):153111.

[42] Gogurla N, Mondal SP, Sinha AK, et al. Transparent and flexible resistive switching memory devices with a very high ON/OFF ratio using gold nanoparticles embedded in a silk protein matrix. Nanotechnology. 2013;24(34):345202. 23912245 10.1088/0957-4484/24/34/345202

[43] Uenuma M, Ban T, Okamoto N, et al. Memristive nanoparticles formed using a biotemplate. RSC Adv. 2013;3(39):18044–18048.

[44] Kim S-J, Hwang B, Lee J-S. Control of gold nanoparticle–protein aggregates in albumen matrix for configurable switching devices. Adv Mater Interfaces. 2018;5(9):1800086.

[45] Wu W, Han S-T, Venkatesh S, et al. Biodegradable skin-inspired nonvolatile resistive switching memory based on gold nanoparticles embedded alkali lignin. Org Electron. 2018;59:382–388.

[46] Chang Y-C, Lee C-J, Wang L-W, et al. Highly uniform resistive switching properties of solution-processed silver-embedded gelatin thin film. Small. 2018;14(13):1703888. 10.1002/smll.201703888 29450966

[47] Raeis Hosseini N, Lee J-S. Resistive switching memory based on bioinspired natural solid polymer electrolytes. ACS Nano. 2015;9(1):419–426. 25513838 10.1021/nn5055909

[48] Hosseini NR, Lee J-S. Biocompatible and flexible chitosan-based resistive switching memory with magnesium electrodes. Adv Funct Mater. 2015;25(35):5586–5592.

[49] Strobel C, Sandner T, Strehle S. Resistive switching memory based on silver-doped chitosan thin films. MRS Adv. 2018;3(33):1943–1948.

[50] Abbas Y, Dugasani SR, Raza MT, et al. The observation of resistive switching characteristics using transparent and biocompatible Cu2^+^-doped salmon DNA composite thin film. Nanotechnology. 2019;30(33):335203. 31026860 10.1088/1361-6528/ab1cfd

[51] Chu H-L, Chiu S-C, Sung C-F, et al. Programmable redox state of the nickel ion chain in DNA. Nano Lett. 2014;14(2):1026–1031. 24456092 10.1021/nl404601s

[52] Pan C, Ji Y, Xiao N, et al. Coexistence of grain-boundaries-assisted bipolar and threshold resistive switching in multilayer hexagonal boron nitride. Adv Funct Mater. 2017;27(10):1604811.

[53] Yagati AK, Kim S-U, Lee T, et al. Recombinant azurin-CdSe/ZnS hybrid structures for nanoscale resistive random access memory device. Biosens Bioelectron. 2017;90:23–30. 27871046 10.1016/j.bios.2016.11.037

[54] Murgunde BK, Rabinal MK. Solution processed bilayer junction of silk fibroin and semiconductor quantum dots as multilevel memristor devices. Org Electron. 2017;48:276–284.

[55] Vallabhapurapu S, Rohom A, Chaure NB, et al. Bistable resistive memory behavior in gelatin-CdTe quantum dot composite film. AIP Conf Proc. 2018;1953(1):030271.

[56] Lv Z, Wang Y, Chen Z, et al. Phototunable biomemory based on light-mediated charge trap. Adv Sci. 2018;5(9):1800714. 10.1002/advs.201800714 PMC6145401 30250806

[57] Yoon J, Mohammadniaei M, Choi HK, et al. Resistive switching biodevice composed of MoS_2_-DNA heterolayer on the gold electrode. Appl Surf Sci. 2019;478:134–141.

[58] Liu T, Wu W, Liao K-N, et al. Fabrication of carboxymethyl cellulose and graphene oxide bio-nanocomposites for flexible nonvolatile resistive switching memory devices. Carbohydr Polym. 2019;214:213–220. 30925991 10.1016/j.carbpol.2019.03.040

[59] Wang M, Wang W, Leow WR, et al. Enhancing the matrix addressing of flexible sensory arrays by a highly nonlinear threshold switch. Adv Mater. 2018;30(33):1802516. 10.1002/adma.201802516 29971867

[60] Barbaro M, Bonfiglio A, Raffo L, et al. Fully electronic DNA hybridization detection by a standard CMOS biochip. Sens Actuators B. 2006;118(1):41–46.

[61] Hung C-Y, Tu W-T, Lin Y-T, et al. Optically controlled multiple switching operations of DNA biopolymer devices. J Appl Phys. 2015;118(23):235503.

[62] Jeng H-Y, Yang T-C, Yang L, et al. Non-volatile resistive memory devices based on solution-processed natural DNA biomaterial. Org Electron. 2018;54:216–221.

[63] Kim M-K, Lee J-S. Ultralow power consumption flexible biomemristors. ACS Appl Mater Interfaces. 2018;10(12):10280–10286. 29464944 10.1021/acsami.8b01781

[64] Raeis-Hosseini N, Lee J-S. Controlling the resistive switching behavior in starch-based flexible biomemristors. ACS Appl Mater Interfaces. 2016;8(11):7326–7332. 26919221 10.1021/acsami.6b01559

[65] Han S-T, Peng H, Sun Q, et al. An overview of the development of flexible sensors. Adv Mater. 2017;29(33):1700375. 10.1002/adma.201700375 28671711

[66] Wang C-H, Hsieh C-Y, Hwang J-C. Flexible organic thin-film transistors with silk fibroin as the gate dielectric. Adv Mater. 2011;23(14):1630–1634. 21360778 10.1002/adma.201004071

[67] Irimia-Vladu M, Sariciftcib NS, Bauer S. Exotic materials for bio-organic electronics. J Mater Chem. 2011;21(5):1350–1361.

[68] Hwang B, Lee J-S. Recent advances in memory devices with hybrid materials. Adv Electron Mater. 2018;5(1):1800519.

[69] Zhou L, Mao J, Ren Y, et al. Recent advances of flexible data storage devices based on organic nanoscaled materials. Small. 2018;14(10):1703126. 10.1002/smll.201703126 29377568

[70] Zafara R, Ziaa KM, Tabasuma S, et al. Polysaccharide based bionanocomposites, properties and applications: a review. Int J Boil Macromol. 2016;92:1012–1024. 10.1016/j.ijbiomac.2016.07.102 27481340

[71] Ko Y, Ryu SW, Cho J. Biomolecule nanoparticle-induced nanocomposites with resistive switching nonvolatile memory properties. Appl Surf Sci. 2016;368:36–43.

[72] Zhu B, Wang H, Leow WR, et al. Silk fibroin for flexible electronic devices. Adv Mater. 2016;28(22):4250–4265. 26684370 10.1002/adma.201504276

[73] Stadler P, Oppelt K, Singh TB, et al. Organic field-effect transistors and memory elements using deoxyribonucleic acid (DNA) gate dielectric. Org Electron. 2007;8(6):648–654.

[74] Irimia-Vladu M, Troshin PA, Reisinger M, et al. Biocompatible and biodegradable materials for organic field-effect transistors. Adv Funct Mater. 2010;20(23):4069–4076.

[75] Choi J, Han JS, Hong K, et al. Organic–inorganic hybrid halide perovskites for memories, transistors, and artificial synapses. Adv Mater. 2018;30(42):1704002. 10.1002/adma.201704002 29847692

[76] Kybert NJ, Han GH, Lerner MB, et al. Scalable arrays of chemical vapor sensors based on DNA-decorated graphene. Nano Res. 2014;7(1):95–103.

[77] Ariga K, Nishikawa M, Mori T, et al. Self-assembly as a key player for materials nanoarchitectonics. Sci Technol Adv Mater. 2019;20(1):51–95. 30787960 10.1080/14686996.2018.1553108 PMC6374972

[78] Miura A, Uraoka Y, Fuyuki T, et al. Floating nanodot gate memory fabrication with biomineralized nanodot as charge storage node. J Appl Phys. 2008;103(7):074503.

[79] Miura A, Tsukamoto R, Yoshii S, et al. Non-volatile flash memory with discrete bionanodot floating gate assembled by protein template. Nanotechnology. 2018;19(25):255201. 10.1088/0957-4484/19/25/255201 21828646

[80] Bera S, Mondal SP, Naskar D, et al. Flexible and transparent nanocrystal floating gate memory devices using silk protein. Org Electron. 2014;15(8):1767–1772.

[81] Ordinario DD, Phan L, Dyke YV, et al. Photochemical doping of protonic transistors from a cephalopod protein. Chem Mater. 2016;28(11):3703–3710.

[82] Chen Y-S, Hong M-Y, Huang GS. A protein transistor made of an antibody molecule and two gold nanoparticles. Nat Nanotechnol. 2012;7(3):197–203. 22367097 10.1038/nnano.2012.7

[83] Lv Z, Chen M, Qian F, et al. Mimicking neuroplasticity in a hybrid biopolymer transistor by dual modes modulation. Adv Funct Mater. 2019;29(31):1902374.

[84] Kim C-H, Bhak G, Lee J. Controlled charge trapping and retention in large-area monodisperse protein metal-nanoparticle conjugates. ACS Appl Mater Interfaces. 2016;8(19):11898–11903. 27144458 10.1021/acsami.6b02268

[85] Bhak G, Lee J, Kim C-H, et al. High-density single-layer coating of gold nanoparticles onto multiple substrates by using an intrinsically disordered protein of α-synuclein for nanoapplications. ACS Appl Mater Interfaces. 2017;9(10):8519–8532. 28248091 10.1021/acsami.6b16411

[86] Zhuang X, Huang W, Yang X, et al. Biocompatible/degradable silk fibroin: poly(vinylalcohol)-blended dielectric layer towards high-performance organic field-effect transistor. Nanoscale Res Lett. 2016;11(1):439. 27709560 10.1186/s11671-016-1660-x PMC5052155

[87] Chiu Y-C, Otsuka I, Halila S, et al. High-performance nonvolatile transistor memories of pentacence using the green electrets of sugar-based block copolymers and their supramolecules. Adv Funct Mater. 2014;24(27):4240–4249.

[88] Sun H-S, Chiu Y-C, Lee W-Y, et al. Synthesis of oligosaccharide-based block copolymers with pendent π-conjugated oligofluorene moieties and their electrical device applications. Macromolecules. 2015;48(12):3907–3917.

[89] Liu R, Zhu LQ, Wang W, et al. Biodegradable oxide synaptic transistors gated by a biopolymer electrolyte. J Mater Chem. 2016;4(33):7744–7750.

[90] Feng P, Du P, Wan C, et al. Proton conducting graphene oxide/chitosan composite electrolytes as gate dielectrics for new-concept devices. Sci Rep. 2016;6:34065. 27688042 10.1038/srep34065 PMC5043185

[91] Du B-W, Hu S-Y, Singh R, et al. Eco-friendly and biodegradable biopolymer chitosan/Y_2_O_3_ composite materials in flexible organic thin-film transistors. Materials. 2017;10(9):1026. 10.3390/ma10091026 PMC5615681 28869517

[92] Liu H, Xun D. Junctionless thin-film transistors gated by an H_3_PO_4_-incorporated chitosan proton conductor. J Nanosci Nanotechnol. 2014;18(4):2910–2913. 10.1166/jnn.2018.14399 29442973

[93] Green NS, Norton ML. Interactions of DNA with graphene and sensing applications of graphene field-effect transistor devices: a review. Anal Chim Acta. 2015;853(1):127–142. 25467454 10.1016/j.aca.2014.10.023

[94] Kajisa T, Sakata T. Glucose-responsive hydrogel electrode for biocompatible glucose transistor. Sci Technol Adv Mater. 2017;18(1):26–33. 28179956 10.1080/14686996.2016.1257344 PMC5256429

[95] Lin T-W, Hsieh P-J, Lin C-L, et al. Label-free detection of protein-protein interactions using a calmodulin-modified nanowire transistor. Proc Natl Acad Sci. 2010;107(3):1047–1052. 20080536 10.1073/pnas.0910243107 PMC2824270

[96] Lim C-M, Lee I-K, Lee KJ, et al. Improved sensing characteristics of dual-gate transistor sensor using silicon nanowire arrays defined by nanoimprint lithography. Sci Technol Adv Mater. 2017;18(1):17–25. 28179955 10.1080/14686996.2016.1253409 PMC5256244

[97] Wang Y, Lv Z, Chen J, et al. Photonic synapses based on inorganic perovskite quantum dots for neuromorphic computing. Adv Mater. 2018;30(38):1802883. 10.1002/adma.201802883 30063261

[98] Zhong Y-N, Wang T, Gao X, et al. Synapse-like organic thin film memristors. Adv Funct Mater. 2018;28(22):1800854.

[99] Yang R, Huang H-M, Hong Q-H, et al. Synaptic suppression triplet-STDP learning rule realized in second-order memristors. Adv Funct Mater. 2018;28(5):1704455.

[100] Patel M, Kim H-S, Park -H-H, et al. Silver nanowires-templated metal oxide for broadband Schottky photodetector. Appl Phy Lett. 2016;108(14):141904.

[101] Yan X, Zhang L, Chen H, et al. Graphene oxide quantum dots based memristors with progressive conduction tuning for artificial synaptic learning. Adv Funct Mater. 2018;28(40):1803728.

[102] Wang Y, Yang J, Ye W, et al. Near-infrared-irradiation-mediated synaptic behavior from tunable charge-trapping dynamics. Adv Electron Mater. 2019;5:1900765.

[103] Byun J, Cho H, Wolf C, et al. Efficient visible quasi-2D perovskite light-emitting diodes. Adv Mater. 2016;28(34):7515–7520. 27334788 10.1002/adma.201601369

[104] Wang Y, Liu E, Gao A, et al. Negative photoconductance in van der waals heterostructure-based floating gate phototransistor. ACS Nano. 2018;12(9):9513–9520. 30118592 10.1021/acsnano.8b04885

[105] Wang Y, Yang J, Wang Z, et al. Near-infrared annihilation of conductive filaments in quasiplane MoSe_2_/Bi2Se_3_ nanosheets for mimicking heterosynaptic plasticity. Small. 2019;15(7):1805431. 10.1002/smll.201805431 30653280

[106] Aimi J, Lo C-T, Wu H-C, et al. Phthalocyanine-cored star-shaped polystyrene for nano floating gate in nonvolatile organic transistor memory device. Adv Electron Mater. 2016;2(2):1500300.

[107] Li B, Liu Y, Wan C, et al. Mediating short-term plasticity in an artificial memristive synapse by the orientation of silica mesopores. Adv Mater. 2018;30(16):1706395. 10.1002/adma.201706395 29544021

[108] Kim M-K, Lee J-S. Short-term plasticity and long-term potentiation in artificial biosynapses with diffusive dynamics. ACS Nano. 2018;12(2):1680–1687. 29357225 10.1021/acsnano.7b08331

[109] Liu YH, Zhu LQ, Feng P, et al. Freestanding artificial synapses based on laterally proton-coupled transistors on chitosan membranes. Adv Mater. 2015;27(37):5599–5604. 26426725 10.1002/adma.201502719

[110] Kuo -C-C, Lin C-H, Chen W-C. Morphology and photophysical properties of light-emitting electrospun nanofibers prepared from poly(fluorene) derivative/PMMA blends. Macromolecules. 2007;40(19):6959–6966.

[111] Nishizawa M. Soft, wet and ionic microelectrode systems. Bull Chem Soc Jpn. 2018;91:1141–1149.

[112] Stauss S, Honma I. Biocompatible batteries-materials and chemistry, fabrication, applications, and future prospects. Bull Chem Soc Jpn. 2018;91:492–505.

